# Cognitive stimulation in the workplace, plasma proteins, and risk of dementia: three analyses of population cohort studies

**DOI:** 10.1136/bmj.n1804

**Published:** 2021-08-19

**Authors:** Mika Kivimäki, Keenan A Walker, Jaana Pentti, Solja T Nyberg, Nina Mars, Jussi Vahtera, Sakari B Suominen, Tea Lallukka, Ossi Rahkonen, Olli Pietiläinen, Aki Koskinen, Ari Väänänen, Jatinderpal K Kalsi, Marcel Goldberg, Marie Zins, Lars Alfredsson, Peter J M Westerholm, Anders Knutsson, Töres Theorell, Jenni Ervasti, Tuula Oksanen, Pyry N Sipilä, Adam G Tabak, Jane E Ferrie, Stephen A Williams, Gill Livingston, Rebecca F Gottesman, Archana Singh-Manoux, Henrik Zetterberg, Joni V Lindbohm

**Affiliations:** 1 Department of Epidemiology and Public Health, University College London, London, UK; 2 Clinicum, Faculty of Medicine, University of Helsinki, Helsinki, Finland; 3 Finnish Institute of Occupational Health, Helsinki, Finland; 4 Department of Neurology, The Johns Hopkins University, Baltimore, MD, USA; 5 Laboratory of Behavioral Neuroscience, National Institute on Aging, Intramural Research Program, Baltimore, MD, USA; 6 Department of Public Health, University of Turku, Turku, Finland; 7 Institute for Molecular Medicine Finland, HiLIFE, University of Helsinki, Helsinki, Finland; 8 Centre for Population Health Research, University of Turku and Turku University Hospital, Turku, Finland; 9 School of Health Science, University of Skövde, Skövde, Sweden; 10 Inserm UMS 011, Population-Based Epidemiological Cohorts Unit, Villejuif, France; 11 Université de Paris, INSERM U1153, Epidemiology of Ageing and Neurodegenerative Diseases, Paris, France; 12 Institute of Environmental Medicine, Karolinska Institutet, Stockholm, Sweden; 13 Centre for Occupational and Environmental Medicine, Region Stockholm, Stockholm, Sweden; 14 Department of Medical Sciences, Uppsala University, Uppsala, Sweden; 15 Department of Health Sciences, Mid Sweden University, Sundsvall, Sweden; 16 Stress Research Institute, Stockholm University, Stockholm, Sweden; 17 Institute of Public Health and Clinical Nutrition, University of Eastern Finland, Kuopio, Finland; 18 Department of Internal Medicine and Oncology and Department of Public Health, Semmelweis University, Budapest, Hungary; 19 Bristol Medical School, University of Bristol, Bristol, UK; 20 SomaLogic, Boulder, CO, USA; 21 Division of Psychiatry, University College London, London, UK; 22 Camden and Islington NHS Foundation Trust, London, UK; 23 Department of Neurodegenerative Disease and UK Dementia Research Institute, University College London, London, UK; 24 Department of Psychiatry and Neurochemistry, Institute of Neuroscience and Physiology, The Sahlgrenska Academy, University of Gothenburg, and Clinical Neurochemistry Laboratory, Sahlgrenska University Hospital, Mölndal, Sweden

## Abstract

**Objectives:**

To examine the association between cognitively stimulating work and subsequent risk of dementia and to identify protein pathways for this association.

**Design:**

Multicohort study with three sets of analyses.

**Setting:**

United Kingdom, Europe, and the United States.

**Participants:**

Three associations were examined: cognitive stimulation and dementia risk in 107 896 participants from seven population based prospective cohort studies from the IPD-Work consortium (individual participant data meta-analysis in working populations); cognitive stimulation and proteins in a random sample of 2261 participants from one cohort study; and proteins and dementia risk in 13 656 participants from two cohort studies.

**Main outcome measures:**

Cognitive stimulation was measured at baseline using standard questionnaire instruments on active versus passive jobs and at baseline and over time using a job exposure matrix indicator. 4953 proteins in plasma samples were scanned. Follow-up of incident dementia varied between 13.7 to 30.1 years depending on the cohort. People with dementia were identified through linked electronic health records and repeated clinical examinations.

**Results:**

During 1.8 million person years at risk, 1143 people with dementia were recorded. The risk of dementia was found to be lower for participants with high compared with low cognitive stimulation at work (crude incidence of dementia per 10 000 person years 4.8 in the high stimulation group and 7.3 in the low stimulation group, age and sex adjusted hazard ratio 0.77, 95% confidence interval 0.65 to 0.92, heterogeneity in cohort specific estimates I^2^=0%, P=0.99). This association was robust to additional adjustment for education, risk factors for dementia in adulthood (smoking, heavy alcohol consumption, physical inactivity, job strain, obesity, hypertension, and prevalent diabetes at baseline), and cardiometabolic diseases (diabetes, coronary heart disease, stroke) before dementia diagnosis (fully adjusted hazard ratio 0.82, 95% confidence interval 0.68 to 0.98). The risk of dementia was also observed during the first 10 years of follow-up (hazard ratio 0.60, 95% confidence interval 0.37 to 0.95) and from year 10 onwards (0.79, 0.66 to 0.95) and replicated using a repeated job exposure matrix indicator of cognitive stimulation (hazard ratio per 1 standard deviation increase 0.77, 95% confidence interval 0.69 to 0.86). In analysis controlling for multiple testing, higher cognitive stimulation at work was associated with lower levels of proteins that inhibit central nervous system axonogenesis and synaptogenesis: slit homologue 2 (SLIT2, fully adjusted β −0.34, P<0.001), carbohydrate sulfotransferase 12 (CHSTC, fully adjusted β −0.33, P<0.001), and peptidyl-glycine α-amidating monooxygenase (AMD, fully adjusted β −0.32, P<0.001). These proteins were associated with increased dementia risk, with the fully adjusted hazard ratio per 1 SD being 1.16 (95% confidence interval 1.05 to 1.28) for SLIT2, 1.13 (1.00 to 1.27) for CHSTC, and 1.04 (0.97 to 1.13) for AMD.

**Conclusions:**

The risk of dementia in old age was found to be lower in people with cognitively stimulating jobs than in those with non-stimulating jobs. The findings that cognitive stimulation is associated with lower levels of plasma proteins that potentially inhibit axonogenesis and synaptogenesis and increase the risk of dementia might provide clues to underlying biological mechanisms.

## Introduction

Cognitive stimulation has been hypothesised to help preserve cognitive function and decrease the risk of dementia in old age.^1^ Studies to date that have focused on cognitive stimulation in adulthood, however, have been small and with insufficient control for confounding factors and have failed to produce compelling evidence of benefits.^2^ ^3^ Trials, typically based on relatively small sample sizes and short term interventions, have reported inconsistent results.^4^ ^5^ ^6^ Most of the recent long term cohort studies have suggested that leisure time cognitive activity does not reduce the risk of dementia either.^7^ ^8^ ^9^ A progressive reduction occurs in cognitive activities before dementia onset, but this seems to be because the gradual onset of dementia results in inactivity rather than lower cognitive activity being related to dementia.^7^ ^8^

It is unclear whether the reason for modest findings is that the decrease in brain plasticity with age prevents cognitive activities across adult life from conferring protection against dementia, or, in the case of interventions, that the cognitive stimulation studied has not been intensive or engaging enough to preserve cognitive function. Work roles might be useful in the study of these associations. Exposure to cognitive stimulation at work can extend over decades and amount to tens of thousands of hours, so this stimulation lasts considerably longer than cognitive interventions or, typically, cognitively stimulating hobbies. According to the demand-control model, cognitively stimulating “active” jobs include demanding tasks and high job decision latitude (also known as job control).^10^ ^11^ Non-stimulating “passive” jobs are those with low demands and lack of job control. The combination of high demands and low control, in turn, characterises stressful work or job strain, which might be a risk rather than a protective factor for dementia.^12^ ^13^

In this report from the IPD-Work consortium (individual participant data meta-analysis in working populations),^14^ a large ongoing multicohort study of work and health, we examined the association between cognitively stimulating work and subsequent risk of dementia, controlling for established dementia risk factors and job strain. To identify potential biological pathways for this association, we performed a data driven analysis of 4953 plasma proteins, measured using slow off-rate modified aptamers.^15^ Plasma proteins are relevant targets for mechanistic study because they are affected by environmental exposures and serve many functions in regeneration, degeneration, and disease.^15^ ^16^ ^17^

## Methods

### Study populations

Established at the Four Centres meeting in London in 2008, the IPD-Work consortium is a collaborative research project of 13 European cohort studies, which aims to estimate associations between work related factors and chronic diseases, disability, and mortality. To meet this goal, the consortium uses predefined exposure definitions (to minimise selective reporting) and large pooled datasets (to allow confirmation of findings across subgroups and, in the case of null findings, to show and publish absence of associations convincingly).

**Fig 1:**
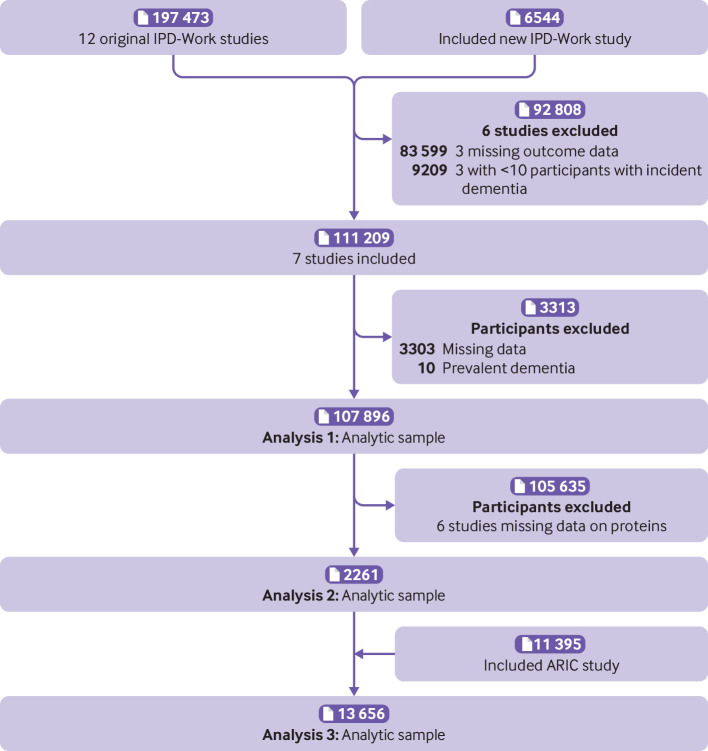
Selection of cohort studies to analyse three associations: cognitive stimulation-dementia (analysis 1), cognitive stimulation-proteins (analysis 2), and proteins-dementia (analysis 3). IPD-Work=individual participant data meta-analysis in working populations; ARIC=Atherosclerosis Risk in Communities

Seven of the 13 eligible cohort studies had relevant data to examine the association between cognitive stimulation at work and dementia incidence (analysis 1, see supplementary figure): the Finnish Public Sector study (FPS); GAZEL study, France; Health and Social Support (HeSSup) study, Helsinki Health Study (HHS), and Still Working study, Finland; the Whitehall II study, UK; and the Works, Lipids, and Fibrinogen (WOLF) Stockholm and Norrland studies, Sweden (fig 1). These studies comprised 107 896 men and women who were free of dementia at baseline (1986 to 2002), when cognitive stimulation and covariates were assessed. Follow-up for dementia was through to end of 2017.

To explore biological plausibility in a data driven analysis (analysis 2, see supplementary figure), we assessed 4953 proteins in plasma from a random sample of 2261 participants in one of the IPD-Work cohorts (the Whitehall II study)^15^ and examined associations between cognitive stimulation and plasma proteins (fig 1). Cognitive stimulation at work was assessed in 1991-93, and blood samples for the assessment of plasma protein were taken in 1997-99. The participants were free of dementia at the assessment of cognitive stimulation and plasma proteins.

In analysis 3 (see supplementary figure), we examined associations between proteins and dementia. As a complementary dataset to Whitehall II with follow-up for dementia until October 2019, we included a non-IPD-Work cohort, the multiethnic Atherosclerosis Risk in Communities (ARIC) study with 11 395 participants (fig 1).^18^ In ARIC, blood samples for protein analyses were drawn in 1993-95 and dementia follow-up was through to end of 2017. Cognitive stimulation at work was not measured in ARIC.

Table 1 summarises the study designs, participant numbers, and outcome ascertainment methods. Supplementary etables 1-8 show the characteristics of the cohorts.

**Table 1:** Study populations in analyses on associations between cognitive stimulation, plasma proteins, and dementia

Analysis and cohorts	Baseline	Total No	Mean (SD) follow-up (years)	No with dementia	Method of dementia ascertainment	Protein assessment
**Cognitive stimulation-dementia (analysis 1)**						
GAZEL	1997	11 362	13.7 (1.4)	14	Self-reports, deaths	–
WOLF	1992-95	10 368	14.1 (2.3)	25	Hospital admissions, deaths	–
HeSSup	1998	15 534	14.8 (1.3)	50	Hospital admissions, prescriptions, deaths	–
HHS	2000-02	6544	14.4 (1.8)	43	Hospital admissions, prescriptions, deaths	–
Whitehall II	1991-93	7475	22.1 (3.4)	183	Hospital admissions, deaths	–
FPS	2000-02	47 448	15.5 (1.6)	313	Hospital admissions, prescriptions, deaths	–
Still Working	1986	9165	30.1 (2.2)	515	Hospital admissions, prescriptions, deaths	–
Pooled total		107 896	16.7 (4.9)	1143		
**Cognitive stimulation-proteins (analysis 2)**						
Whitehall II (random sample):						
Cognitive stimulation	1991-93	2261			–	
Proteins	1997-99	2261	20.4 (3.2)	109	–	Somascan, version 4
**Proteins-dementia (analysis 3)**						
Whitehall II (random sample)	1997-99	2261	20.4 (3.2)	109	Hospital admissions, deaths	Somascan, version 4
ARIC	1993-95	11 395	17.7 (6.1)	1942	Clinical examination, Hospital admissions, deaths	Somascan, version 4
Total		13 656	18.1	2051		

WOLF=Works, Lipids, and Fibrinogen Stockholm and Norrland studies; HeSSup=Health and Social Support study; FPS=Finnish Public Sector study; ARIC=Atherosclerosis Risk in Communities study.

### Measurement of cognitive stimulation at work

We used predefined protocols to assess indicators of cognitive stimulation at work: job demands and job control. A description of the self-administered multi-item measures of these characteristics in each participating IPD-Work study and data harmonisation are available elsewhere and in the supplementary file (pp 13-14).^19^ Briefly, to minimise investigator bias, we validated and harmonised the job demand and control measures across participating cohort studies before extracting data for dementia, with investigators masked to outcome information. Questions in the job demands and job control scales had Likert-type response formats. We computed mean response scores for job demand items and for job control items for each participant. High job demand was defined as having a job demand score that was greater than the study specific median score; we defined high job control as having a job control score that was higher than or equal to the study specific median score. These categorisations are the originals and most used. We used the dichotomised measures to construct three categories of cognitive stimulation at work along the active-passive work dimension of Karasek’s demand-control model^10^ ^11^ in which both high demands and high control indicate higher stimulation. We defined low cognitive stimulation at work as low demands and low control, medium cognitive stimulation as high control and low demands or high demands and low control, and high cognitive stimulation as high demands and high control. The supplementary file (pp 13-14, efigure 1) provides a detailed description of the demand-control model.

To obtain an alternative continuous longitudinal measure unaffected by individual response style for supplementary analyses, we used a job exposure matrix indicator of cognitive stimulation that captures any changes of job and changes in level of stimulation over time. This measure was available for participants in the largest cohort study, the FPS. Data on participants’ occupational titles were obtained from Statistics Finland at baseline in 2000 and at follow-up in 2005, 2010, and 2015 and were categorised according to the International Standard Classification of Occupations (ISCO-88) by the International Labour Organisation (www.ilo.org/public/english/bureau/stat/isco/isco88/index.htm, accessed 3 July 2021). Using the three digit ISCO codes, we identified a total of 87 different occupations with at least 20 participants to determine the job axis for the job exposure matrix. The exposure axis was constructed by calculating the proportion of workers with high cognitive stimulation at work based on all responses within the same occupation. This occupation based exposure value was then assigned to each participant in the occupation (ranging from 0% for booking clerks to 62% for city mayors and other top administrators) in 2000, 2005, 2010, and 2015 or until the participant retired or was censored owing to dementia or death.

### Covariates

Covariates were established dementia risk factors across the life course, as defined by the 2020 *Lancet* Commission on Dementia.^1^ These included participants’ age and sex, obtained from national or employers’ registers or self-reported. Education, a measure of cognitive stimulation in childhood, was self-reported (GAZEL, HHS, Still Working, Whitehall, WOLF, HeSSup, ARIC), or obtained from national registers (FPS).^20^ Education was categorised into low (primary or lower secondary), intermediate (higher secondary), and high (tertiary qualification, college, or university) levels.

Risk factors in adulthood included smoking (current versus former or never smoker), alcohol consumption (heavy (>14 units/week in women, >21 units/week in men versus other; one unit is approximately equivalent to 10 g of ethanol), physical activity (high versus low), obesity (body mass index ≥30 versus <30), hypertension (yes versus no), and prevalent diabetes (yes versus no).^21^ ^22^ ^23^ ^24^ ^25^ ^26^ Job strain (yes versus no) was measured with questions from the validated job content or demand-control questionnaire,^19^ and, as in other IPD-Work studies, was defined as high demands and low control, with all other combinations denoting no job strain.^14^ ^27^

Because people with cardiometabolic disease have increased risk of dementia, covariates also included diabetes (diagnostic codes E10 and E11 in the international classification of diseases, 10th revision; ICD-10), coronary heart disease (non-fatal myocardial infarction, ICD-10 codes I21–I22, or coronary death recorded as ICD-10 I20–I25), and stroke (ICD-10 codes I60, I61, I63, I64), including both prevalent cases at baseline and incident cases between baseline and before dementia diagnosis, assessed using linked records.

When possible we used additional covariates, available for specific cohorts only. Analyses of a longitudinal job exposure matrix indicator of cognitive stimulation (analysis 1) were based on the FPS study, with data on several additional dementia risk factors: social isolation (yes or no), depression (ICD-10 F32, F33), traumatic brain injury (ICD-10 S06), and atrial fibrillation (ICD-10 I48); the last three were identified from records of hospital admissions before dementia diagnosis. In protein analyses (analyses 2 and 3), we additionally adjusted effect estimates for ethnicity (white versus non-white) because more than 20% of the ARIC participants were non-white. In protein analyses based on the Whitehall II subcohort, APOE genotype (0, 1, or 2 of ε4 risk alleles), a strong predictor of dementia, was an additional covariate. Further information about additional covariates is available in the supplementary file (pp 14-15, etables 9-11).

### Measurement of plasma proteins

Proteins were analysed using the SomaScan version 4 assay (Somalogic, CO).^15^ The analyses used plasma EDTA samples collected in 1997-99 (Whitehall II) or 1993-95 (ARIC) and stored at−80°C. Earlier studies and the supplementary file (p 16) describe in detail the performance of the SomaScan assay and the modified aptamer binding.^28^ ^29^ ^30^ Briefly, the assay uses a mix of thousands of slow off-rate modified aptamers (SOMAmers). The aptamers bind to proteins in participants’ plasma samples and the specificity is ensured by a two step process analogous to a conventional immunoassay. The specificity of the aptamer reagents is good and has been tested in several ways.^15^ Median intra-assay and inter-assay coefficients of variation for SomaScan version 4 are about 5%, and assay sensitivity is comparable to that of typical immunoassays, with a median lower limit of detection in the femtomolar range.^28^

### Follow-up for dementia

For the seven IPD-Work studies, including Whitehall II and its subcohort, we extracted data on dementia status at follow-up from hospital admissions records and death registries with any mention of dementia in the diagnosis, with or without reimbursements for medical treatment for dementia (Anatomical Therapeutic Chemical code N06D; table 1).^20^ Electronic records included exact date of diagnosis, death, or entitlement to reimbursement, and follow-up duration was measured as the difference between date of baseline examination and date of diagnosis, death, or entitlement to reimbursement. Although ascertainment of dementia from electronic health records underestimates prevalence, it has been shown to be a valid method in the study of associations between risk factors and dementia.^31^ ^32^ ^33^ ICD-10 codes for all cause dementia were F00, F01, F02, F03, G30, and G31, with earlier ICD codes converted to ICD-10 codes (supplementary file p16). Codes F00 and G30 were used to define Alzheimer’s disease.

In the ARIC study, adjudicated people with dementia were primarily identified using data from ARIC clinic examinations conducted at visits 5 (2011-13) and 6 (2016-17). This included a neuropsychological battery using standardised protocols, with scores converted to z scores to assess change over time. Dementia was identified based on important decline on a serial cognitive battery, poor current test performance on a comprehensive neuropsychological battery, and impairments in activities of daily living based on informant rating on the clinical dementia rating scale and the functional activities questionnaire. A committee of clinicians then adjudicated participants with suspected dementia based on available cognitive and functional data. For participants who could not attend visits 5 or 6, those with dementia were identified through telephone cognitive assessment, plus surveillance of hospital discharge and death certificate codes related to dementia as well as screening during annual and semi-annual follow-up calls. Dementia suspected in participants who had died was identified through informant interviews.

### Statistical analysis

We analysed the data in three parts (table 1). Cognitive stimulation at work was treated as a three level categorical variable, with low stimulation as the reference. The longitudinal job exposure matrix indicator of cognitive stimulation was analysed as a continuous exposure variable. In all three analyses, in multivariable models we adjusted effect estimates for age, sex, established dementia risk factors in childhood (education) and adulthood (smoking, heavy alcohol consumption, physical inactivity, job strain, obesity, hypertension, prevalent diabetes), and cardiometabolic diseases before dementia (ie, prevalent and incident diabetes, coronary heart disease, and stroke, treated as time dependent covariates). Before analysis, we imputed missing values in covariates using multiple imputation by substantive model compatible fully conditional specification.^34^ With data from all study variables, cohort indicator, and baseline year, 30 imputed datasets were created for the pooled dataset, Whitehall II random sample, and ARIC cohort.

#### Analysis 1 (cognitive stimulation-dementia)

To examine associations of cognitively stimulating work with dementia risk, we followed each participant from the date of cognitive stimulation assessment to the first record of dementia, death, or the end of follow-up. We used a two stage approach, including study specific analyses with Cox regression in the first stage and pooling the study specific estimates with random effects meta-analysis in the second. To estimate heterogeneity among the study specific estimates, we calculated I^2^ (higher values denoting greater heterogeneity).

To examine the robustness of the findings, we performed separate analyses for men and women, younger (<60 years at baseline) and older participants (≥60 years at baseline), and an alternative, repeatedly measured job exposure matrix indicator of cognitive stimulation at work, entered into the model as a time dependent exposure. We also performed the analysis separately for incident dementia during the first 10 years of follow-up (when assessment of cognitively stimulating work might become inaccurate in the preclinical or prodromal stage of dementia) and incident dementia from year 10 onwards in those without a dementia diagnosis at year 10. The assumption in analyses separating the assessment of cognitively stimulating work and dementia diagnosis by at least 10 years is that the cognitive stimulation-dementia association is less likely to be biased owing to reverse causation. To examine whether the association between cognitive stimulation and dementia depended on the method of dementia ascertainment, we stratified analyses by ascertainment method with the following categories: primary and specialised medical care (ascertainment based on self-report, prescription records, hospital admission, and death records) versus specialised medical care only (hospital admissions and death records). We also examined the association of cognitive stimulation with early onset (diagnosis aged <65) and late onset (diagnosis aged ≥65) dementia, Alzheimer’s disease, and non-Alzheimer’s disease dementia.

To address potential survival bias, we conducted a Fine and Gray competing risk analysis, with dementia and death as outcomes.^35^ In addition to the standard set of covariates (ie, age, sex, education, risk factors in adulthood, and cardiometabolic disease before dementia), we included cohort indicator as a covariate in multivariable adjusted analyses based on pooled data. Further covariates, available for analyses of the longitudinal job exposure matrix indicator of cognitive stimulation, included social isolation, depression, traumatic brain injury, and atrial fibrillation at baseline.

#### Analysis 2 (cognitive stimulation-proteins)

The distribution of many of the plasma proteins was skewed. We applied rank based inverse normal transformation to achieve normal distributions. Logistic regression was used to study the associations of proteins with high versus low cognitive stimulation and medium versus low cognitive stimulation and were expressed as odds ratios per 1 standard deviation higher level of protein. Tests of statistical significance were corrected for multiple comparison using the Bonferroni method for 4953 tests (P<1.0×10^−5^). In addition to adjustments for the standard set of covariates, we adjusted the effect estimates for ethnicity and *APOE* genotype.

#### Analysis 3 (proteins-dementia)

Cox proportional hazards models were used to study associations between proteins that survived Bonferroni correction in analysis 2 and dementia. In the two cohorts, hazard ratios were computed for 1 standard deviation higher protein level, adjusting for age, sex, ethnicity, and the standard set of covariates. We conducted a Fine and Gray competing risk analysis to address potential survival bias.^35^ Cohort specific effect estimates were pooled using fixed effect meta-analysis.

#### Post hoc analyses

To examine the effect of cognitive stimulation across the life course, we created a life course measure of cognitive stimulation by combining education and cognitive stimulation at work into a single variable, including the categories low education-low cognitive stimulation (reference), low education-high cognitive stimulation, high education-low cognitive stimulation, and high education-high cognitive stimulation. Here the low category included the low and medium categories of the original education and cognitive stimulation measures. We used Cox regression to assess the age, sex, and cohort adjusted association between cognitive stimulation across the life course and incident dementia.

We used SAS (version 9.4) to analyse associations in each cohort and in the pooled data in analysis 1, Stata (version 16.1) for multiple imputations, meta-analyses combining cohort specific estimates, and protein analyses. The supplementary file (pp 26-33) shows the statistical code.

### Patient and public involvement

This is a secondary analysis of pre-existing datasets. No patients were involved in setting the present research question, setting the outcome measures, or developing plans for recruitment, design, or implementation of the study. No patients were asked to advise on the interpretation, but we have received feedback from a patient reviewer of *The BMJ*. The dissemination plan targets a wide audience, including members of the public, patients, health professionals, and experts in the specialty through various channels: written communication, events and conferences, networks, and social media.

## Results

Overall, 2488 (2.3%) of 110 394 eligible participants who had missing data for cognitive stimulation or incident dementia and 10 (<0.1%) with a diagnosis of Alzheimer’s disease or other dementia before the study baseline were excluded from analyses. Of 107 896 participants included in the cognitive stimulation-dementia analysis (table 2), 45 080 (41.8%) were men and 62 816 (58.2%) were women, with a mean age of 44.6 (SD 9.5) years, at baseline. Of the participants, 29 243 (27.1%) had low cognitive stimulation at work, 50 724 (47.0%) had medium stimulation, and 27 929 (25.9%) had high stimulation.

**Table 2:** Characteristics of participants from pooled sample

Characteristic	No (%)
Sex:	
Men	45 080 (41.8)
Women	62 816 (58.2)
Mean (SD) age (years)	44.6 (9.5)
Age group (years):	
<60	105 065 (97.4)
≥60	2831 (2.6)
Cognitive stimulation at work:	
Low	29 243 (27.1)
Medium	50 724 (47.0)
High	27 929 (25.9)
Education:	
Low	26 036 (25.1)
Intermediate	40 723 (39.2)
High	37 171 (35.8)
Current smoking:	
No	81 821 (79.1)
Yes	21 580 (20.9)
Heavy alcohol intake:	
No	95 043 (89.7)
Yes	10 950 (10.3)
Physical inactivity:	
No	82 030 (77.8)
Yes	23 358 (22.2)
Obesity (BMI ≥30):	
No	86 611 (89.2)
Yes	10 507 (10.8)
Hypertension:	
No	91 858 (86.9)
Yes	13 878 (13.1)
Prevalent diabetes:	
No	105 199 (97.5)
Yes	2680 (2.5)
Job strain:	
No	90 766 (84.1)
Yes	17 130 (15.9)
Prevalent or incident diabetes:	
No	99 970 (92.8)
Yes	7909 (7.3)
Prevalent or incident coronary heart disease:	
No	105 540 (97.8)
Yes	2356 (2.2)
Prevalent or incident stroke:	
No	105 523 (97.8)
Yes	2373 (2.2)

Supplementary eTables 1-7 present the characteristics of participants by cohort.

BMI=body mass index.

### Cognitive stimulation-dementia risk

**Fig 2:**
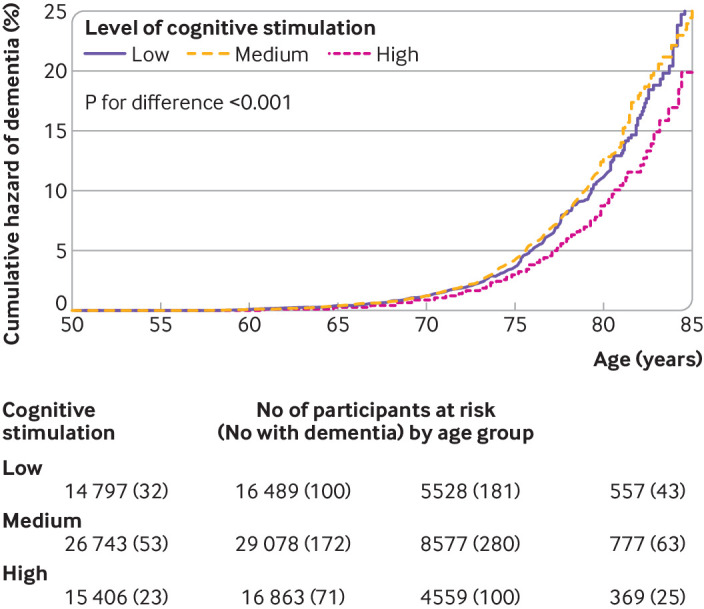
Crude cumulative hazard of dementia by age and level of cognitive stimulation at work

Mean follow-up for dementia varied between 13.7 and 30.1 years depending on the cohort and was 16.7 (SD 4.9) years in the total sample. During 1 801 863 person years at risk, 1143 participants had a diagnosis of dementia between the ages of 42 and 93 years, mean 71.2 (SD 7.9) years (supplementary efigure 2). Cumulative hazards of dementia by age and level of cognitive stimulation at baseline showed a separation in dementia occurrence between high and low stimulation groups such that the cumulative incidence of dementia seen in participants who had high cognitive stimulation at work was observed at a younger age in those who had low stimulation at work (fig 2). For example, the cumulative incidence at age 75 in the high stimulation group (3.1%) was already observed at age 74 in the low stimulation group, and the incidence at age 80 (8.8%) was already observed at age 78.3, respectively. No difference in dementia was observed between participants with medium and low cognitive stimulation.

**Fig 3:**
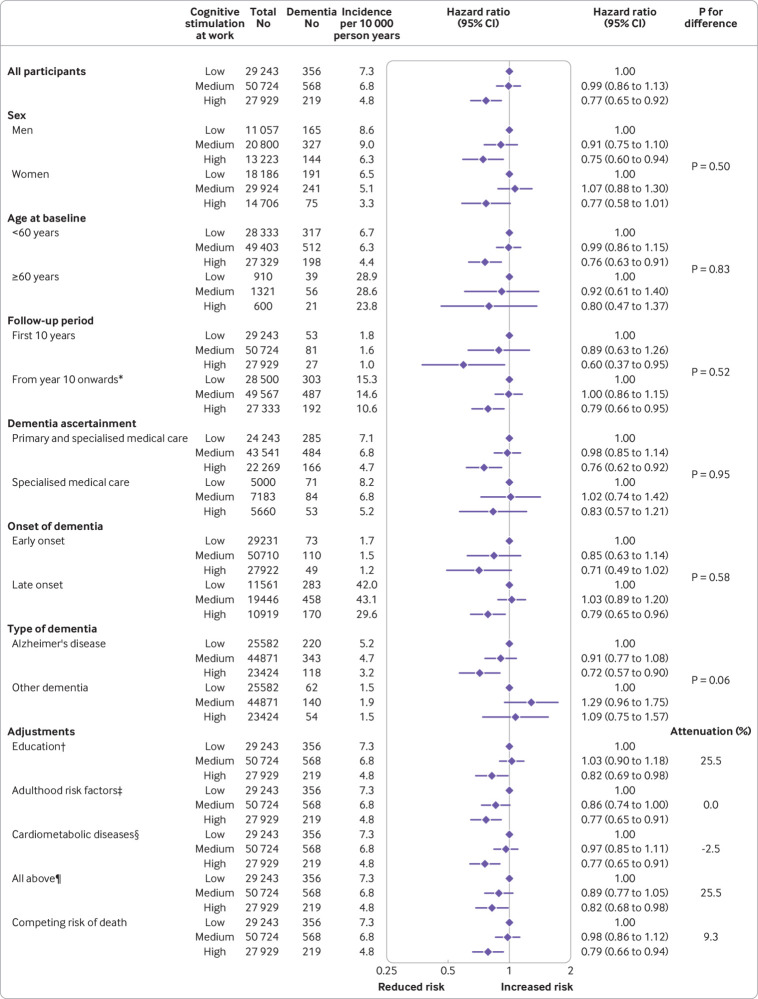
Association of cognitive stimulation at work with incident dementia in total sample, subgroups, by study follow-up and dementia type, and in relation to adjustments (analysis 1). *Follow-up started 10 years after baseline and the analysis included only those participants without a diagnosis of dementia by that time. †Adjusted for age, sex, cohort, and education. ‡Adjusted for age, sex, cohort, and smoking, alcohol consumption, physical inactivity, job strain, obesity, hypertension, and prevalent diabetes at baseline. §Adjusted for age, sex, and cohort, and diabetes, coronary heart disease, and stroke (prevalent at baseline and incident between baseline and dementia diagnosis). ¶Adjusted for age, sex, education, risk factors in adulthood, and cardiometabolic diseases

Meta-analysis of the seven cohort studies confirmed the association between high cognitive stimulation and lower risk of dementia in later life (fig 3). Incidence of dementia per 10 000 person years was 7.3 in the low cognitive stimulation group and 4.8 in the high stimulation group, the corresponding age and sex adjusted hazard ratio being 0.77 (95% confidence interval 0.65 to 0.92). This finding showed no significant heterogeneity across the cohorts (I^2^=0%, P=0.99) and the pattern of results was similar in the five cohorts with more than 25 participants with dementia (supplementary efigure 3). The association between higher cognitive stimulation and lower dementia risk was slightly weaker than that between education and dementia (hazard ratio 0.66, 95% confidence interval 0.55 to 0.79, supplementary etable 12), but the association was statistically significant and robust in analyses stratified by sex, age, and method of dementia ascertainment, before and after exclusion of participants with early onset dementia to minimise reverse causation, and before and after adjustment for established dementia risk factors in childhood and adulthood and the competing risk of death. The association was not mediated by prevalent or incident cardiometabolic diseases, although these diseases were related to increased dementia risk (age and sex adjusted hazard ratio 1.45, 95% confidence interval 1.23 to 1.71 for diabetes, 1.36, 1.08 to 1.72 for coronary heart disease, and 1.90, 1.51 to 2.38 for stroke). There was an indication that the cognitive stimulation-dementia association was attributable to Alzheimer’s diseases rather than to other dementias (P=0.06).

In the FPS study, the mean for the job exposure matrix indicator of cognitive stimulation was 22.7% (SD 12.7%, range 0-62%). Repeating the analyses with this longitudinal, repeated measure of cognitive stimulation replicated the main findings (age and sex adjusted hazard ratio per 1 SD higher stimulation 0.77, 95% confidence interval 0.69 to 0.86) (supplementary etable 13). This association remained after adjustment for established dementia risk factors, competing risk of death, and additional covariates, such as social isolation, depression, traumatic brain injury, and atrial fibrillation.

### Cognitive stimulation, plasma proteins, and dementia risk

In the Whitehall subcohort with protein assessments, data from 4953 plasma proteins were available for 2261 participants (supplementary etable 10). None of the participants had dementia at the assessment of cognitive stimulation and proteins. After correction for multiple testing and adjustment for age, sex, and ethnicity, levels of the following six proteins were found to be significantly lower among participants with high compared with low cognitive stimulation: pulmonary surfactant associated protein D (SP-D), slit homologue 2 protein (SLIT2), hexokinase 2 (HXK2), carbohydrate sulfotransferase 12 (CHSTC), peptidyl-glycine α-amidating monooxygenase (AMD), and neutrophil cytosol factor 1 (NCF-1) (table 3 and supplementary efigure 4). No association between medium versus low cognitive stimulation at work and plasma proteins reached Bonferroni adjusted significance. The inverse associations between high cognitive stimulation and low levels of the six proteins were independent of established dementia risk factors, cardiometabolic diseases, and *APOE* genotype (supplementary etable 14).

**Table 3:** Proteins associated with cognitive stimulation in 2261 participants with plasma proteins available in Whitehall subcohort after controlling for multiple testing (analysis 2)

Protein	High *v* low cognitive stimulation			Medium *v* low cognitive stimulation	
	β (SE)*	P value		β (SE)*	P value
Pulmonary surfactant associated protein D (SP-D)	−0.32 (0.06)	<0.001		-0.17 (0.06)	0.002
Slit homologue 2 protein (SLIT2)	−0.30 (0.06)	<0.001		−0.17 (0.06)	0.002
Hexokinase 2 (HXK2)	−0.28 (0.06)	<0.001		−0.13 (0.06)	0.019
Carbohydrate sulfotransferase 12 (CHSTC)	−0.28 (0.06)	<0.001		−0.08 (0.06)	0.18
Peptidyl-glycine α-amidating monooxygenase (AMD)	−0.28 (0.06)	<0.001		−0.12 (0.06)	0.03
Neutrophil cytosol factor 1 (NCF-1)	−0.27 (0.06)	<0.001		−0.06 (0.06)	0.28

* Per 1 standard deviation higher protein level. Only statistically significant associations after adjustment for age, sex, ethnicity, and Bonferroni correction for multiple testing (P<1.0×10^−5^) are shown for high versus low cognitive stimulation.

Analysis of the association between the six plasma proteins and dementia was based on data from the Whitehall subcohort and the ARIC study. Mean follow-up for dementia after the assessment of proteins was 20.4 (SD 3.2) years in the Whitehall subcohort and 17.7 (SD 6.1) in ARIC, giving rise to 106 and 1942 people with dementia, respectively. In age, sex, and ethnicity adjusted analysis of combined data from the Whitehall subcohort and ARIC, higher levels of SLIT2, CHSTC, and AMD were associated with increased dementia risk, or, conversely, lower protein levels were associated with lower dementia risk (table 4). The effect estimates did not materially change after adjustments (supplementary etables 15 and 16). No consistent association with dementia was seen for HXK2, SP-D, or NFC-1.

**Table 4:** Association of six plasma proteins with incident dementia in two cohort studies (analysis 3)

Protein and cohort	Total No	No with dementia		Adjusted hazard ratio (95% CI) for dementia*	Directionally consistent and significant†
SLIT2:					
Whitehall	2261	109		1.19 (0.97 to 1.45)	
ARIC	11 395	1942		1.12 (1.00 to 1.26)	Yes
Both	13 656	2051		1.14 (1.03 to 1.25)	
CHSTC:					
Whitehall	2261	109		1.08 (0.90 to 1.31)	
ARIC	11 395	1942		1.22 (1.05 to 1.41)	Yes
Both	13 656	2051		1.17 (1.04 to 1.31)	
AMD:					
Whitehall	2261	109		1.11 (0.92 to 1.34)	
ARIC	11 395	1942		1.07 (0.99 to 1.16)	Yes
Both	13 656	2051		1.08 (1.00 to 1.16)	
HXK2:					
Whitehall	2261	109		1.09 (0.91 to 1.31)	
ARIC	11 395	1943		1.03 (0.92 to 1.15)	No
Both	13 656	2051		1.05 (0.95 to 1.15)	
SP-D:					
Whitehall	2261	109		1.16 (0.95 to 1.40)	
ARIC	11 395	1943		0.98 (0.93 to 1.04)	No
Both	13 656	2051		0.99 (0.94 to 1.05)	
NCF-1:					
Whitehall	2261	109		0.94 (0.78 to 1.13)	
ARIC	11 395	1943		1.25 (1.12 to 1.41)	No
Both	13 656	1944		1.16 (1.05 to 1.28)	

AMD=peptidyl-glycine α-amidating monooxygenase; ARIC=Atherosclerosis Risk in Communities; CHSTC=carbohydrate sulphotransferase 12; HXK2=hexokinase 2; NCF-1=neutrophil cytosol factor 1; SLIT2=slit homologue 2 protein; SP-D=pulmonary surfactant associated protein D.

* Adjusted for age, sex, and ethnicity.

† Hazard ratio >1 in both cohort studies and P<0.05 in pooled analysis.

### Post hoc analysis of cognitive stimulation in childhood and adulthood

**Fig 4:**
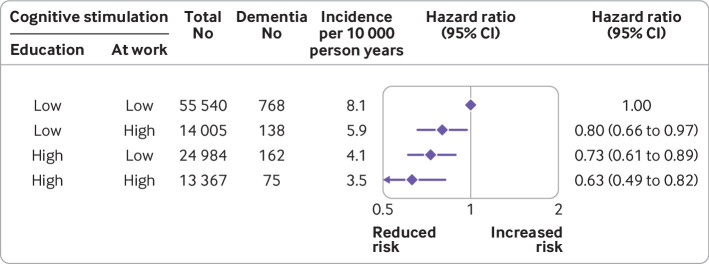
Association of cognitive stimulation over the life course with incident dementia (post hoc analysis)

A dose-response association was observed between cognitive stimulation over the life course and dementia such that cognitive stimulation in both childhood (education) and adulthood (cognitive stimulation at work) were related to a reduced risk of dementia (fig 4). The hazard ratio for high education and high cognitive stimulation at work compared with low education and low cognitive stimulation at work was 0.63 (95% confidence interval 0.49 to 0.82), whereas those for categories with high education or high cognitive stimulation at work, but not both, were 0.80 (0.66 to 0.97) and 0.73 (0.61 to 0.89), respectively.

## Discussion

In analysis of individual level data from more than 100 000 people, those who worked in cognitively stimulating jobs showed a lower risk of dementia than those working in non-stimulating jobs. This finding was robust to adjustments for a range of established dementia risk factors in childhood and adulthood, cardiometabolic diseases, and the competing risk of death. The association did not differ between men and women or those younger and older than 60 and did not depend on the measure of cognitive stimulation at work or the method of dementia ascertainment, but there was an indication that the cognitive stimulation-dementia association was stronger for Alzheimer’s disease than for other dementias. Lower dementia incidence was observed even when 10 years or more separated the assessment of cognitive stimulation and the dementia diagnosis, suggesting that the findings were unlikely to be biased due to reverse causation. In data driven proteome-wide analyses we identified three plasma proteins linked to both cognitive stimulation in adulthood and dementia, providing clues to underlying biological mechanisms.

### Comparison with other studies

Cognitive decline characterises midlife and old age^36^ and it has been suggested that cognitive stimulation might be one measure to slow this neurodegenerative process.^1^ Trial evidence on this possibility is, however, inconsistent. Long term cohort studies on leisure time cognitive activity have reported null findings, and observational studies on work complexity, intellectual engagement, and active jobs have been based on small samples, post hoc exposure measures, and insufficient control for confounding factors.^2^ ^3^ ^7^ ^8^ ^9^ ^37^ ^38^ ^39^ Thus, both the World Health Organization dementia review^40^ and the 2020 *Lancet* Commission on Dementia Prevention, Intervention, and Care^1^ rated the evidence on the association between cognitive stimulation in adulthood and dementia as inconclusive and the quality of studies as very low to low. The 2020 *Lancet* Commission did not include cognitive stimulation in adulthood in the list of targets for dementia prevention.^1^

We hypothesised that methodological limitations and insufficient duration of cognitive stimulation in trial data might have resulted in these inconclusive findings. With the largest multicohort sample to date, rigorous confounder adjustments, and assessment of a cognitive stimulation exposure that is more long lasting than the cognitive interventions or cognitively stimulating hobbies examined in most previous studies, we obtained what we consider to be strong observational evidence for an association between cognitive stimulation in adulthood and dementia. This association was confirmed in a post hoc analysis that combined education and work related cognitive stimulation into a single variable. We found that higher cognitive stimulation in both childhood, as indicated by higher educational attainment, and adulthood, based on work characteristics, were associated with lower dementia risk in a stepwise dose-response manner. Together with mendelian randomisation analyses showing delayed dementia onset in those with higher education,^41^ these findings support the benefits of cognitive stimulation across the life course (see supplementary efigure 5 for a schematic presentation of this hypothesis).

### Meaning of the study

The relative risk for low compared with high cognitive stimulation was 1.3, which is comparable to established dementia risk factors, such as high versus moderate alcohol consumption (relative risk 1.2) and low versus high physical activity (1.4) but is smaller than those for education (1.6), diabetes (1.5), smoking (1.6), hypertension (1.6), and obesity (1.6).^1^ Our observational evidence on cognitive stimulation in adulthood is consistent with the “use it or lose it” principle. However, the robustness of the observed association to adjustment for a wide range of dementia risk factors, cardiometabolic diseases, and the competing risk of death raises the question of underlying mechanisms: if known risk factors do not drive the association, what then are the mediators?

We performed an exploratory and data driven plasma proteome analysis of biological mechanisms underlying the association between cognitive stimulation at work and risk of dementia. We identified three proteins, SLIT2, AMD, and CHSTC for which plasma levels were found to be significantly lower among participants with cognitive stimulation at work, whereas high levels of these proteins were found to be associated with increased dementia incidence. Experimental findings from stem cells, tissue cultures, and animal models suggest that SLIT2 and AMD are involved in central nervous system related mechanisms that inhibit neuron projection axonogenesis, affect axon guidance, and increase nerve degeneration.^42^ ^43^ ^44^ ^45^ CHSTC has been linked to processes that inhibit axonogenesis and cause collapse of axonal growth cones and loss of synaptic connection.^46^ ^47^ ^48^ In light of this molecular evidence and given that dysregulated axonogenesis and synaptic disorganisation are the hallmarks of Alzheimer’s disease,^49^ our observations on plasma proteins are consistent with the hypothesis that protection against neurodegeneration might mediate the favourable effects of cognitive stimulation in adulthood. Further research is needed to confirm or refute this hypothesis.

### Strengths and limitations of this study

The main strengths of the study are its large sample size, which reduces the risk of type 1 error, the availability of nearly 5000 plasma proteins to explore potential underlying mechanisms, and the use of multicohort analyses to provide a certain degree of validation for the main findings. Our study was based on preplanned exposure definitions and subgroup analyses, reducing the risk of reporting bias. We were also able to reduce the risk of reverse causation bias by excluding all participants with dementia during the first 10 years of follow-up. In main or sensitivity analyses, we adjusted effect estimates for 10 of the 12 risk factors established for dementia by the 2020 *Lancet* Commission (ie, education, smoking, heavy alcohol consumption, physical inactivity, obesity, hypertension, diabetes, social isolation, depression, and traumatic brain injury).^1^ The remaining two risk factors are hearing loss and air pollution. Hearing loss is, however, assumed to result in cognitive decline through reduced cognitive stimulation.^1^ Thus, hearing loss represents an alternative measure of cognitive stimulation rather than a confounding factor. We had no data on air pollution.

This study has several weaknesses. Because we used non-randomised observational data, we cannot draw conclusions about causality and cannot exclude residual confounding (eg, by childhood IQ) as an alternative explanation for our findings. As we focused on working age populations at baseline, follow-up was right truncated with a relatively low mean age at dementia diagnosis (mean 71, range 43-93). This precluded assessment of lifelong effects in relation to cognitive stimulation. Rather than having a single dataset with complete information, we used a sequential approach in which we established the presence of the associations in separate analyses and separate cohorts: cognitive stimulation-dementia, cognitive stimulation-proteins, and proteins-dementia. The reason for this piecewise design was that complete data for all these variables were available only for a subgroup in one of the studies, Whitehall II. The sample size of this subgroup was insufficient for a formal mediation analysis of the role of proteins in the association between cognitive stimulation and dementia risk. Main analyses were based on a single self-reported measurement of cognitive stimulation at work. However, consistent results with an alternative, longitudinal job exposure matrix indicator of cognitive stimulation suggest this is an unlikely source of major bias in this study. Except for one cohort in which dementia was diagnosed in repeated clinical examinations (ARIC), ascertainment of dementia was based on linkage to electronic health records, mostly hospital admissions. Although this had the advantage of providing data for everyone recruited to the study, it misses participants with milder dementia and is not the gold standard method for assessing dementia subtypes.

### Generalisability, implications, and future challenges

These results seem to be generalisable across different populations. Although we used cohort studies from different settings (civil servants, public sector employees, forestry workers), little heterogeneity existed in the cohort specific estimates of dementia (heterogeneity I^2^=0%). In addition, the association between cognitive stimulation at work and lower dementia risk was observed across subgroups; men and women and those younger and older than 60 at baseline. These robust findings might be important for policy, suggesting that cognitively stimulating work for all will be important for future brain health. However, further research capturing the full range of occupations and countries outside Europe is needed to confirm the generalisability of our findings.

Our findings on proteins were consistent across the two cohorts with relevant data but owing to smaller numbers and less precise estimates these results need further validation in other studies. If confirmed, the associations might provide clues to search for mechanisms underlying long term risk of dementia in humans. As the proteins identified in this study are associated with a wide range of biological processes beyond neural influences, their effects should be comprehensively examined.

### Conclusions

This multicohort study of more than 100 000 participants suggests that people with cognitively stimulating jobs have a lower risk of dementia in old age than those with non-stimulating jobs. A possible mechanism for this association is the finding that cognitive stimulation is associated with lower levels of plasma proteins that might inhibit axonogenesis and synaptogenesis and increase dementia risk in old age.

**What is already known on this topic**

Cognitive stimulation is assumed to prevent or postpone the onset of dementia, but trials, typically based on relatively small sample sizes and short term interventions, have reported inconsistent results, and most recent long term cohort studies have suggested that leisure time cognitive activity does not reduce risk of dementia
One reason for modest findings could be that the cognitive stimulation in these studies has not been intensive or engaging enough to preserve cognitive function
Exposure to cognitive stimulation at work lasts considerably longer than cognitive interventions, or, typically, cognitively stimulating hobbies; however, studies of cognitive stimulation at work to date have been small with insufficient control for confounding factors and have failed to produce compelling evidence of benefits

**What this study adds**

In this multicohort study of 107 896 participants, the risk of dementia in old age was found to be lower in individuals with cognitively stimulating jobs than in those with non-stimulating jobs
This finding was robust to adjustments for education, established dementia risk factors in adulthood, and the competing risk of death
Cognitive stimulation was also associated with lower levels of plasma proteins that might inhibit axonogenesis and synaptogenesis and increase dementia risk

## Data Availability

Pre-existing access policies for individual level data for each of the participating cohort studies specify that research data requests can be submitted to each steering committee; these will be promptly reviewed for confidentiality, data protection issues, or intellectual property restrictions and will not unreasonably be refused. Code for the statistical analyses of this paper is provided in the supplementary file (pp 26-33).

## Web extra

Extra material supplied by authors

Supplementary information: eTables 1-16, eFigures 1-5, and statistical code

Supplementary information: figure showing details of the three analyses

## Notes

Contributors: MK, JP, STN, and JVL generated the hypothesis and designed the study. JV and SBS (Health and Social Support study), JE and TO (Finnish Public Sector study), TL, OR, and OP (Helsinki Health study), AK and AV (Still Working study), MG and MZ (GAZEL study), LA, PJMW, AK, and TT (WOLF-Stockholm and WOLF-Norland studies), JKK, JEF, and AS-M (Whitehall II study), and KAW and RFG (Atherosclerosis Risk in Communities study, ARIC) were responsible for cohort data. SAW was responsible for the protein assessments. JP performed pooled analyses on cognitive stimulation and dementia. JVL conducted imputed analyses in pooled data and, with NM, analysed protein data from the Whitehall study. KAW supervised protein analyses in the ARIC study. GL, PNS, and JEF contributed to expertise in dementia risk factors. HZ and AGT contributed to dementia and metabolic biology expertise. MK wrote the first draft of the report and JEF helped in revising the text. All authors discussed results and commented on the manuscript. MK is the guarantor. The corresponding author attests that all listed authors meet authorship criteria and that no others meeting the criteria have been omitted.

The lead author affirms that this manuscript is an honest, accurate, and transparent account of the study being reported; that no important aspects of the study have been omitted; and that any discrepancies from the study as planned (and, if relevant, registered) have been explained.

Dissemination to participants and related patient and public communities: The dissemination plan targets a wide audience, including members of the public, patients, patient and public communities, health professionals, and experts in the specialty through various channels: written communication, events and conferences, networks, and social media.

Provenance and peer review: Not commissioned; externally peer reviewed.
