# Supplementary material for: Cognitive stimulation in the workplace, plasma proteins, and risk of dementia: three analyses of population cohort studies
Source: BMJ. 2021 Aug 19;374:n1804. doi: 10.1136/bmj.n1804 (PMC8372196; doi:10.1136/bmj.n1804)
Supplement: Supplementary file 1 — Supplementary information: eTables 1-16, eFigures 1-5, and statistical code [file kivm064249.ww.pdf]

Supplementary information

	Page
The IPD-consortium and participating study cohorts	2
eTable 1. Characteristics of participants from the GAZEL study	4
eTable 2. Characteristics of participants from the WOLF study	6
eTable 3. Characteristics of participants from the HeSSup study	7
eTable 4. Characteristics of participants from the Helsinki Health Study	8
eTable 5. Characteristics of participants from the Still Working study	9
eTable 6. Characteristics of participants from the FPS study	10
eTable 7. Characteristics of participants from the Whitehall II study and subcohort	13
eTable 8. Characteristics of the participants from the ARIC study	15
Demand-control model of cognitive stimulation and job strain at work	14
eFigure 1. Definition of cognitive stimulation based on the demand-control model	14
Measurement of additional covariates	14
eTable 9. Additional covariates in FPS	15
eTable 10. Ethnicity in the Whitehall II subcohort and the ARIC study	15
eTable 11. APOE genotype in the Whitehall II subcohort	15
Measurement of plasma proteins	16
Conversion of earlier ICD-codes for dementia to ICD-10 codes	16
eFigure 2. Distribution of age at dementia diagnosis in the main analysis	16
eFigure 3. Meta-analysis of the 7 cohort studies on the association between cognitive stimulation at work and risk of dementia	17
eTable 12. Association of education with incident dementia in total sample	18
eTable 13. Association between cognitive stimulation based on job exposure matrix and incident dementia in relation to adjustments and additional covariates	19
eFigure 4. Manhattan plot of plasma proteins and high/medium vs low cognitive stimulation	20
eTable 14. Multivariable adjusted associations of 3 plasma proteins with cognitive stimulation	21
eTable 15. Multivariable adjusted associations of 3 plasma proteins with incident dementia by cohort	22
eTable 16. Summary estimates of associations between 3 plasma proteins and incident dementia in relation to adjustments	24
eFigure 5. Cognitive function across the life course according to independent effects of education and adulthood cognitive stimulation and an education-confounded effect of adulthood cognitive stimulation	25
Statistical code	26
References	33

The IPD-Work consortium

Established at the Four Centres meeting in London in 2008, the Individual-Participant-Data meta-analysis in Working populations (IPD-Work) consortium is a collaborative research project of European cohort studies with the aim to estimate reliably the associations of work-related factors with chronic diseases, disability, and mortality. The original participating cohort studies were:

- Whitehall II, UK;
- GAZEL, France;
- Belstress, Belgium;
- Netherlands Working Conditions Survey (NWCS) and
- Permanent Onderzoek Leefsituatie (POLS), Netherlands;
- Intervention Project on Absence and Well-being (IPAW),
- Copenhagen Psychosocial Questionnaire version (COPSOQ-I) and
- Danish Work Environment Cohort Study (DWECS), Denmark;
- Work, Lipids and Fibrinogen-Stockholm and
- Work, Lipids and Fibrinogen-Norrland, Sweden (in combination WOLF);
- Still working study,
- Finnish Public Sector study (FPS), and
- Health and Social Support (HeSSup) study, Finland.

Subsequently, the Helsinki Health Study, Finland (HHS, an occupational cohort study set up in collaboration with the Whitehall II study) has joined the consortium.

As described in the two background papers of the consortium, published research assessing work-related risk factors for chronic conditions had been inconsistent and subject to post hoc decision making, publication bias and reverse causation bias.[1 2] To strengthen evidence, the aim of the IPD-Work consortium is to use pre-defined and harmonized exposure definitions (to minimize selective reporting) and large pooled datasets (to allow confirmation of findings across subgroups and, in case of null finding, to show and publish absence of associations convincingly).

In agreement with these principles, measurements of job demands and job control were validated and harmonized across the IPD-Work cohort studies before extracting outcome data.[3] In addition to the present paper on dementia, the IPD-Work consortium has used these pre-defined measures in studies of diabetes, coronary heart disease, stroke, site-specific cancers, and respiratory diseases.[4-10]

The work of the IPD-Work consortium has been supported by: the Wellcome Trust Collaborative Award in Science (221854/Z/20/Z), Medical Research Council (MR/S011676/1), and Economic and Social Science Research Council (ESRC, ES/J023299/1), UK; National Institute on Aging (NIH), US (R01AG062553); the European Union New OSH ERA ('New and Emerging Risks in Occupational Safety and Health') Research Programme; NordForsk, the Nordic Programme on Health and Welfare (75021); Academy of Finland (132944, 311492); Finnish Work Environment Fund (112253, 115421, 190424), and Helsinki Institute of Life Science (H970), Finland.

Description of participating study cohorts

Atherosclerosis Risk in Communities (ARIC)[11] is a prospective cohort study of men and women recruited from four communities: Forsyth County, North Carolina; Jackson, Mississippi; eight northern suburbs of Minneapolis, Minnesota; and Washington County, Maryland, US. A total of 15,792 participants received an extensive examination, including medical, social, and demographic data and were re-examined every three years with the first screen (baseline) occurring in 1987-89. Data on proteins, based on blood samples drawn in the 1993-95 examination, and dementia incidence were available for 11,395 participants, the analytic sample of the present study. The ARIC study has been approved by the Institutional Review Boards of all participating institutions. Unlike this study, all the remaining cohorts are part of the IPD-Work consortium. The study was approved by each site's institutional review board (at The Johns Hopkins University, Wake Forest University, University of Mississippi Medical Center, and University of Minnesota), and written informed consent was signed by all participants (and proxies, when required)

Finnish Public Sector study (FPS)[12] is a prospective cohort study comprising the entire public sector personnel of 10 towns (municipalities) and 21 hospitals in the same geographical areas[65]. Participants,

who were recruited from employers' records in 2000-2002, were individuals who had been employed in the study organisations for at least six months prior to data collection. 48 592 individuals (9 337 men and 39 255 women aged 17 to 65) responded to the questionnaire. Of these, 47,448 had data on cognitive stimulation at work and were eligible for our meta-analyses and 47,455 had data on the job exposure matrix index of cognitive stimulation. Ethical approval was obtained from the ethics committee of the Finnish Institute of Occupational Health.

GAZEL[13] is a prospective cohort study of 20 625 employees (15,011 men and 5,614 women) of France's national gas and electricity company, Electricité de France-Gaz de France (EDF-GDF). Since the study baseline in 1989, when the participants were aged 35–50 years, they have been posted an annual follow-up questionnaire to collect data on health, lifestyle, individual, familial, social, and occupational factors. Cognitive stimulation at work was measured in Gazel in 1997, which we treated as a baseline year for our analyses. 11 448 individuals participated that year and 11,362 of them had data on cognitive stimulation at work and were eligible for our meta-analysis. The GAZEL study received approval from the national commission overseeing ethical data collection in France (Commission Nationale Informatique et Liberté).

Health and Social Support (HeSSup)[14] is a prospective cohort study of a stratified random sample of the Finnish population in the following four age groups: 20–24, 30–34, 40–44, and 50–54[68]. The participants were identified from the Finnish population register and posted an invitation to participate, along with a baseline questionnaire, in 1998 (4). Job strain was measured in 1998 and of the 25 898 individuals who responded to the questionnaire, 15 534 were in employment and had data on cognitive stimulation at work and were thus eligible for our meta-analyses. The Turku University Central Hospital Ethics Committee approved the study.

Helsinki Health Study (HHS)[15] is a prospective cohort study comprising all employees of the City of Helsinki, who turned 40, 45, 50, 55, or 60 years in 2000-2002. We included in this study all participants who responded to the baseline survey (n=8960, response rate 67%) and provided an informed written consent to combine their survey responses on cognitive stimulation with retrospective and prospective register-based follow-up data on different diseases and mortality (n=6544). Ethical approvals for this study were obtained from the ethics committees of the health authorities of the City of Helsinki, and the Department of Public Health, University of Helsinki.

Still Working Study[16] is an ongoing prospective cohort study. In 1986, the employees (n = 12 173) at all Finnish centres of operation of Enso Gutzeit (a forestry products manufacturer) were invited to participate in a questionnaire survey on demographic, psychosocial and health-related factors. At baseline, 9 282 individuals responded, and of these 9 165 had data on cognitive stimulation at work and were eligible for our meta-analyses. The study was approved by the ethics committee of the Finnish Institute of Occupational Health.

The Whitehall II study[17] is a prospective cohort study set up to investigate socioeconomic determinants of health. At study baseline in 1985-1988, 10,308 civil service employees (6,895 men and 3,413 women) aged 35-55 and working in 20 civil service departments in London were invited to participate in the study. Data on cognitive stimulation at work, measured at phase 3 were available for 7475 men of the men and women who were eligible for our meta-analyses. The Whitehall II study protocol was approved by the University College London Medical School committee on the ethics of human research. Written informed consent was obtained at each data collection wave.

WOLF (Work, Lipids, and Fibrinogen).[18 19] WOLF-Stockholm cohort includes participants aged 19–70 and working in companies in Stockholm county and the WOLF Norrland includes participants aged 19-65 working in companies in Jämtland and Västernorrland counties, a total of 10 368 participants with data on cognitive stimulation and dementia. At study baseline the participants underwent a clinical examination and completed a set of health questionnaires. For WOLF Stockholm, the baseline assessment was undertaken at 20 occupational health units between November 1992 and June 1995 and for WOLF Norrland at 13 occupational health service units in 1996-98. The Regional Research Ethics Board in Stockholm, and the ethics committee at Karolinska Institutet, Stockholm, Sweden approved the study. In analyses, data from these cohorts were pooled and WOLF treated as a single study.

Characteristics of participating cohort studies

Sample characteristics and the proportion of missing data for each variable are described in etables 1 to 7 by cohort.

eTable 1. Characteristics of the GAZEL study

Characteristic		N (%) or M (SD)	% of missing data
**Sex**			0.0
	Men	8225 (72.4)	
	Women	3137 (27.6)	
**Age, mean (SD)**		50.3 (3.0)	
**Age group**			0.0
	< 60 years	11362 (100.0)	
	≥ 60 years	0 (0.0)	
**Cognitive stimulation at work**			0.0
	Low	2281 (20.1)	
	Medium	5323 (46.9)	
	High	3758 (33.1)	
**Established risk factors**			
Education			1.7
	Low	7666 (68.6)	
	Intermediate	3509 (31.4)	
	High	0 (0.0)	
Current smoking			0.0
	No	9336 (82.2)	
	Yes	2026 (17.8)	
Heavy alcohol consumption (>14/21 units per week in men and women)			2.7
	No	9029 (81.6)	
	Yes	2031 (18.4)	
Physical inactivity			6.5
	No	6627 (62.4)	
	Yes	4001 (37.7)	
Obesity (BMI ≥ 30 kg/m^2)			0.9
	No	10235 (90.9)	
	Yes	1024 (9.1)	
Hypertension			0.0
	No	9958 (87.6)	
	Yes	1404 (12.4)	
Prevalent diabetes			0.2
	No	10882 (95.9)	
	Yes	463 (4.1)	
**Work characteristics**			
Job strain			0.0

	No	9716 (85.5)	
	Yes	1646 (14.5)	
**Cardiometabolic disease before dementia**			
Prevalent or incident diabetes			0.2
	No	10150 (89.5)	
	Yes	1195 (10.5)	
Prevalent or incident coronary heart disease			0.0
	No	10984 (96.7)	
	Yes	378 (3.3)	
Prevalent or incident stroke			0.0
	No	10976 (96.6)	
	Yes	386 (3.4)	

eTable 2. Characteristics of the Work, Lipids and Fibrinogen (WOLF) Norrland and Stockholm study

Characteristic		N (%) or M (SD)	% of missing data
**Sex**			0.0
	Men	7141 (68.9)	
	Women	3227 (31.1)	
**Age, mean (SD)**		42.7 (10.8)	
**Age group**			0.0
	< 60 years	9838 (94.9)	
	≥ 60 years	530 (5.1)	
**Cognitive stimulation at work**			0.0
	Low	3007 (29.0)	
	Medium	4405 (42.5)	
	High	2956 (28.5)	
**Established risk factors**			
Education			0.2
	Low	2337 (22.6)	
	Intermediate	5275 (51.0)	
	High	2739 (26.5)	
Current smoking			2.4
	No	7808 (77.2)	
	Yes	2310 (22.8)	
Heavy alcohol consumption (>14/21 units per week in men and women)			2.3
	No	9401 (92.8)	
	Yes	733 (7.2)	
Physical inactivity			0.3
	No	7762 (75.1)	
	Yes	2574 (24.9)	
Obesity (BMI ≥ 30 kg/m^2)			0.3
	No	9273 (89.7)	
	Yes	1061 (10.3)	
Hypertension			0.0
	No	8359 (80.6)	
	Yes	2009 (19.4)	
Prevalent diabetes			0.0
	No	10198 (98.4)	
	Yes	170 (1.6)	
**Work characteristics**			
Job strain			0.0
	No	8848 (85.3)	
	Yes	1520 (14.7)	
**Cardiometabolic disease before dementia**			
Prevalent or incident diabetes			0.0
	No	10068 (97.1)	
	Yes	300 (2.9)	
Prevalent or incident coronary heart disease			0.0
	No	10119 (97.6)	
	Yes	249 (2.4)	
Prevalent or incident stroke			0.0
	No	10134 (97.7)	
	Yes	234 (2.3)	

eTable 3. Characteristics of the Health and Social Support (HeSSup) study

Characteristic		N (%) or M (SD)	% of missing data
**Sex**			0.0
	Men	6912 (44.5)	
	Women	8622 (55.5)	
**Age, mean (SD)**		39.6 (10.3)	
**Age group**			0.0
	< 60 years	15534 (100.0)	
	≥ 60 years	0 (0.0)	
**Cognitive stimulation at work**			0.0
	Low	4168 (26.8)	
	Medium	7304 (47.0)	
	High	4062 (26.2)	
**Established risk factors**			
Education			8.3
	Low	3589 (25.2)	
	Intermediate	8373 (58.8)	
	High	2276 (16.0)	
Current smoking			8.1
	No	10510 (73.7)	
	Yes	3759 (26.3)	
Heavy alcohol consumption (>14/21 units per week in men and women)			0.1
	No	13963 (90.0)	
	Yes	1557 (10.0)	
Physical inactivity			0.6
	No	12274 (79.5)	
	Yes	3164 (20.5)	
Obesity (BMI ≥ 30 kg/m^2)			0.5
	No	13929 (90.1)	
	Yes	1529 (9.9)	
Hypertension			0.5
	No	14318 (92.6)	
	Yes	1139 (7.4)	
Prevalent diabetes			0.0
	No	15245 (98.1)	
	Yes	289 (1.9)	
**Work characteristics**			
Job strain			0.0
	No	12823 (82.6)	
	Yes	2711 (17.5)	
**Cardiometabolic disease before dementia**			
Prevalent or incident diabetes			0.0
	No	14606 (94.0)	
	Yes	928 (6.0)	
Prevalent or incident coronary heart disease			0.0
	No	15318 (98.6)	
	Yes	216 (1.4)	
Prevalent or incident stroke			0.0
	No	15318 (98.6)	
	Yes	216 (1.4)	

eTable 4. Characteristics of the Helsinki Health Study (HHS)

Characteristic		N (%) or M (SD)	% of missing data
**Sex**			0.0
	Men	1414 (21.6)	
	Women	5130 (78.4)	
**Age, mean (SD)**		49.4 (6.6)	
**Age group**			0.0
	< 60 years	5742 (87.7)	
	≥ 60 years	802 (12.3)	
**Cognitive stimulation at work**			0.0
	Low	1668 (25.5)	
	Medium	3075 (47.0)	
	High	1801 (27.5)	
**Established risk factors**			
Education			0.1
	Low	925 (14.2)	
	Intermediate	2253 (34.5)	
	High	3358 (51.4)	
Current smoking			4.5
	No	4751 (76.0)	
	Yes	1502 (24.0)	
Heavy alcohol consumption (>14/21 units per week in men and women)			0.5
	No	6211 (95.4)	
	Yes	303 (4.7)	
Physical inactivity			0.6
	No	5194 (79.8)	
	Yes	1313 (20.2)	
Obesity (BMI ≥ 30 kg/m^2)			0.8
	No	5536 (85.3)	
	Yes	955 (14.7)	
Hypertension			0.7
	No	5007 (77.1)	
	Yes	1491 (23.0)	
Prevalent diabetes			0.0
	No	6366 (97.3)	
	Yes	178 (2.7)	
**Work characteristics**			
Job strain			0.0
	No	5451 (83.3)	
	Yes	1093 (16.7)	
**Cardiometabolic disease before dementia**			
Prevalent or incident diabetes			0.0
	No	5918 (90.4)	
	Yes	626 (9.6)	
Prevalent or incident coronary heart disease			0.0
	No	6488 (99.1)	
	Yes	56 (0.9)	
Prevalent or incident stroke			0.0
	No	6457 (98.7)	
	Yes	87 (1.3)	

eTable 5. Characteristics of the Still Working study

Characteristic		N (%) or M (SD)	% of missing data
**Sex**			0.0
	Men	7081 (77.3)	
	Women	2084 (22.7)	
**Age, mean (SD)**		40.9 (9.1)	
**Age group**			0.0
	< 60 years	9086 (99.1)	
	≥ 60 years	79 (0.9)	
**Cognitive stimulation at work**			0.0
	Low	2800 (30.6)	
	Medium	4502 (49.1)	
	High	1863 (20.3)	
**Established risk factors**			
Education			6.5
	Low	4469 (52.2)	
	Intermediate	3733 (43.6)	
	High	365 (4.3)	
Current smoking			1.1
	No	6073 (67.0)	
	Yes	2992 (33.0)	
Heavy alcohol consumption (>14/21 units per week in men and women)			1.4
	No	8607 (95.3)	
	Yes	429 (4.8)	
Physical inactivity			2.1
	No	7221 (80.5)	
	Yes	1748 (19.5)	
Obesity (BMI ≥ 30 kg/m^2)			100.0
	No	-	
	Yes	-	
Hypertension			0.0
	No	8693 (94.9)	
	Yes	472 (5.2)	
Prevalent diabetes			0.0
	No	9079 (99.1)	
	Yes	86 (0.9)	
**Work characteristics**			
Job strain			0.0
	No	7738 (84.4)	
	Yes	1427 (15.6)	
**Cardiometabolic disease before dementia**			
Prevalent or incident diabetes			0.0
	No	8349 (91.1)	
	Yes	816 (8.9)	
Prevalent or incident coronary heart disease			0.0
	No	8646 (94.3)	
	Yes	519 (5.7)	
Prevalent or incident stroke			0.0
	No	8694 (94.9)	
	Yes	471 (5.1)	

eTable 6. Characteristics of the Finnish Public Sector study (FPS)

Characteristic		N (%) or M (SD)	% of missing data
**Sex**			0.0
	Men	9101 (19.2)	
	Women	38347 (80.8)	
**Age, mean (SD)**		44.6 (9.4)	
**Age group**			0.0
	< 60 years	46201 (97.4)	
	≥ 60 years	1247 (2.6)	
**Cognitive stimulation at work**			0.0
	Low	13326 (28.1)	
	Medium	23337 (49.2)	
	High	10785 (22.7)	
**Job exposure matrix indicator of cognitive stimulation***			
	Year 2000 (Baseline, N=47455)	22.7 (12.7)	0.0
	Year 2005 (N=42413)	23.3 (12.9)	0.0
	Year 2010 (N=35095)	24.3 (12.7)	0.0
	Year 2015 (N=27139)	25.4 (12.5)	0.0
**Established risk factors at baseline**			
Education			0.0
	Low	5246 (11.1)	
	Intermediate	16182 (34.1)	
	High	26020 (54.8)	
Current smoking			4.9
	No	37087 (82.2)	
	Yes	8049 (17.8)	
Heavy alcohol consumption			2.5
	No	41558 (89.8)	
	Yes	4702 (10.2)	
Physical inactivity			3.0
	No	37005 (80.4)	
	Yes	9030 (19.6)	
Obesity (BMI ≥ 30 kg/m^2)			2.2
	No	41130 (88.7)	
	Yes	5254 (11.3)	
Hypertension			4.3
	No	39510 (87.0)	
	Yes	5901 (13.0)	
Prevalent diabetes			0.0
	No	46354 (97.7)	
	Yes	1094 (2.3)	
**Work characteristics at baseline**			
Job strain			0.0
	No	39713 (83.7)	
	Yes	7735 (16.3)	
**Cardiometabolic diseases before dementia**			
Prevalent or incident diabetes			0.0
	No	44666 (94.1)	
	Yes	2782 (5.9)	
Prevalent or incident coronary heart disease			0.0
	No	46900 (98.9)	
	Yes	548 (1.2)	
Prevalent or incident stroke			0.0
	No	46617 (98.3)	
	Yes	831 (1.8)	

* Number of participants decreases at follow-up due to retirement, dementia or death, not due to missing data

eTable 7. Characteristics of the Whitehall II study and its subgroup with protein data

Characteristic		Whitehall II (step 1) N (%) or M (SD)	Whitehall II (step 1) % of missing data	Whitehall (subgroup, steps 2 & 3) N (%) or M (SD)	Whitehall (subgroup, steps 2 & 3) % of missing data
**Sex**			0.0		0.0
	Men	5206 (69.7)		1650 (73.0)	
	Women	2269 (30.4)		611 (27.0)	
**Age, mean (SD)**		48.9 (5.8)		56.1 (5.9)	0.0
**Age group**			0.0		
	< 60 years	7302 (97.7)		1554 (68.7)	
	$\geq$ 60 years	173 (2.3)		707 (31.3)	
**Cognitive stimulation at work**			0.0		0.0
	Low	1993 (26.7)		553 (24.5)	
	Medium	2778 (37.2)		949 (42.0)	
	High	2704 (36.2)		759 (33.6)	
**Established risk factors**					
Education			24.9		25.7
	Low	1804 (32.1)		522 (31.1)	
	Intermediate	1398 (24.9)		427 (25.4)	
	High	2413 (43.0)		730 (43.5)	
Current smoking			3.7		6.0
	No	6256 (86.9)		1895 (89.2)	
	Yes	942 (13.1)		230 (10.8)	
Heavy alcohol consumption (>14/21 units per week in men and women)			0.1		2.9
	No	6274 (84.0)		1825 (83.1)	
	Yes	1195 (16.0)		371 (16.9)	
Physical inactivity			0.0		2.7
	No	5947 (79.6)		1806 (82.1)	
	Yes	1528 (20.4)		393 (17.9)	
Obesity (BMI $\geq$ 30 kg/m^2)			3.8		4.3
	No	6508 (90.5)		1967 (90.9)	
	Yes	684 (9.5)		196 (9.1)	
Hypertension			0.0		1.1

	No	6013 (80.4)		1761 (78.8)	
	Yes	1462 (19.6)		474 (21.2)	
Prevalent diabetes			0.0		1.1
	No	7075 (94.7)		2226 (99.6)	
	Yes	400 (5.4)		9 (0.4)	
**Work characteristics**					
Job strain			0.0		0.0
	No	6477 (86.7)		1971 (87.2)	
	Yes	998 (13.4)		290 (12.8)	
**Cardiometabolic disease before dementia**					
Prevalent or incident diabetes			0.0		1.1
	No	6213 (83.1)		1750 (78.3)	
	Yes	1262 (16.9)		485 (21.7)	
Prevalent or incident coronary heart disease			0.0		1.1
	No	7085 (94.8)		2101 (94.0)	
	Yes	390 (5.2)		134 (6.0)	
Prevalent or incident stroke			0.0		1.1
	No	7327 (98.0)		2189 (97.9)	
	Yes	148 (2.0)		46 (2.1)	

eTable 8. Characteristics of the participants in the ARIC study before imputations

Characteristic		N (%) or M (SD)	% of missing data
**Sex**			0
	Men	5190 (45.5%)	
	Women	6205 (54.5%)	
**Age, mean (SD)**		60.1 (5.7)	
**Age group**			0
	< 60 years	5450 (47.8%)	
	≥ 60 years	5945 (52.2%)	
**Ethnicity**			
	White	8991 (78.9)	0
	Non-white	2404 (21.1)	
**Established risk factors**			
Education			0.1
	Low	2311 (20.3%)	
	Intermediate	4813 (42.3%)	
	High	4255 (37.4%)	
Current smoking			0.3
	No	9321 (82%)	
	Yes	2042 (18%)	
Current alcohol use			0.3
	No	5997 (52.8%)	
	Yes	5368 (47.2%)	
Obesity (BMI ≥ 30 kg/m^2)			0.1
	No	7598 (66.8%)	
	Yes	3783 (33.2%)	
Hypertension			0.5
	No	6651 (58.7%)	
	Yes	4686 (41.3%)	
Prevalent diabetes			0.3
	No	9557 (84.1%)	
	Yes	1806 (15.9%)	
**Cardiometabolic disease before dementia**			
Diabetes before dementia			8.4
	No	6344 (60.8%)	
	Yes	4094 (39.2%)	
Coronary heart disease before dementia			0
	No	9375 (82.3%)	
	Yes	2020 (17.7%)	
Stroke before dementia			0.2
	No	10344 (91%)	
	Yes	1029 (9%)	

Demand-control model of cognitive stimulation

The level of cognitive stimulation was defined based on the active-passive dimension, the lowest cognitive stimulation relating to passive jobs in which both job demands and job control are low and the highest cognitive stimulation to active jobs in which both job demands and job control are high (eFigure 2). Medium cognitive stimulation refers to jobs in which job demands are high, but job control low or job demands are low, but job control high.

Karasek describes the active-passive dimension as follows: "…incremental additions to competency are predicted to occur when the challenges of the situation are matched by the individual's skill or control in dealing with a challenge. When job demands and job decision latitude are simultaneously high, we define the job as "active" and hypothesize that it leads to development of new behaviour patterns both on and off the job (Diagonal B toward lower right). The model predicts that jobs at the opposite extreme (defined as "passive job") induce a decline in overall activity and a reduction in general problem-solving activity."[20] (P. 288)

The other dimension in Karasek's model is job strain. This is typically operationalised as a dichotomous variable with high demands and low control referring to job strain and all other combinations of demands and control to no job strain.[20 21]

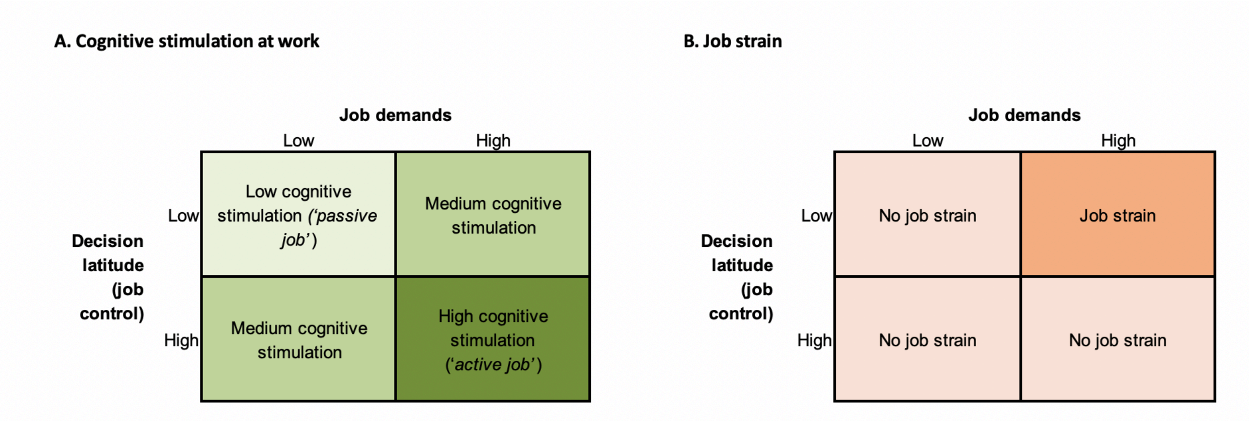

eFigure 1. Definition of cognitive stimulation (A) and job strain (B) based on the Demand-Control Model

<u>Measurement of cognitive stimulation</u>: The assessment of cognitive stimulation at work was based on Karasek's model of two fundamental characteristics of work, job demands and job control (also labelled as job decision latitude).[20] In IPD-Work cohort studies, questions in the job demands and job control scales had Likert-type response formats. Mean response scores for job demand items and for job control items were computed for each participant.[4] We defined high job demands as having a job-demand score that was greater than the study-specific median score; similarly, we defined low job control as having a job control score that was lower than the study-specific median score. These categorisations are the original and most commonly used.

We used dichotomised demand and control measures to construct three categories of cognitive stimulation at work along the active-passive work dimension of Karasek's demand-control model[20 21] in which both high demands and high control indicate higher stimulation. Low cognitive stimulation at work was defined as 'low demands and low control', medium cognitive stimulation as 'high control and low demands' or 'high demands and low control' and high cognitive stimulation as 'high demands and high control'.

Although scale items slightly varied between studies, all versions were strongly related to the original gold standard scales (Pearson correlation coefficients between $r = 0.76$ and $r = 0.98$).[3]

Measurement of additional covariates

Additional covariates in the FPS were: Social isolation (based on an affirmative response to questions on living alone), depression (based on hospital admission records with an ICD-10 code F32 or F33),

traumatic brain injury (based on hospital admission records with an ICD-10 code S06), and atrial fibrillation (based on hospital admission records with an ICD-10 code I48). Social isolation was measured at baseline. We considered hospital admissions both at baseline and during follow-up until dementia diagnosis in dementia cases and the end of follow-up in non-cases. The distribution of these covariates is shown in etable 9.

eTable 9. Additional covariates in FPS

Covariate	N	%
Social isolation		
No	39832	84.8
Yes	7120	15.2
Depression		
No	46169	98.3
Yes	783	1.7
Traumatic brain injury		
No	46552	99.1
Yes	400	0.9
Atrial fibrillation		
No	45827	97.6
Yes	1125	2.4

In the Whitehall-II random sample and ARIC studies, ethnicity was measured by self-report and was categorized into 'white' vs 'non-white'. The distribution of this covariate by cohort is shown in etable 10.

eTable 10. Ethnicity in Whitehall and ARIC studies

	Whitehall II random sample	ARIC
**Ethnicity**, No. (%)		
White	2088 (92.3)	8991 (78.9)
Non-white	173 (7.7)	2404 (21.1)

In the Whitehall-II random sample, for APOE genotyping (0, 1 or 2 of ε4 risk alleles), two TaqMan assays (Rs429358 and Rs7412, Assay-On-Demand, Applied Biosystems) were used and run on a 7900HT analyzer (Applied Biosystems). Genotypes were indicated by the Sequence Detection Software version 2.0 (Applied Biosystems). Each SNP was found to be in Hardy Weinberg Equilibrium. Distribution of APOE genotype in Whitehall II is shown in etable 11.

eTable 11. APOE genotype in the Whitehall II random sample

APOE genotype	N (%)
N of epsilo4 alleles	
0	1432 (73.4)
1	469 (24.1)
2	49 (2.5)

Measurement of plasma proteins

We measured plasma proteins using the SomaScan Assay, version 4.[22] The assay, its performance characteristics and the modified aptamer binding reagents have previously been described[23-25] and the annotated menu for all ~5,000 modified-aptamer binding reagents is available in our previous paper.[22] Briefly, the SomaScan Assay uses a 96-well plate including a mix of thousands of slow off-rate modified aptamers (SOMAmers). The SOMAmers are labelled with biotin and a 5′ fluorophore, photocleavable linker and immobilized on streptavidin-coated beads through biotin–streptavidin interaction. Participants' plasma samples were diluted and added to each well, which resulted in formation of cognate and non-specific SOMAmer-protein complexes. After the unbound proteins were washed away, captured proteins were labelled with biotin. By using ultraviolet light photocleavage, the SOMAmer-protein complexes were released from the beads and incubated in a buffer containing unlabelled polyanionic competitor. This step separates the unspecific, fast off-rate SOMAmer-protein complexes from slow off-rate cognate and thus intended complexes, and can be viewed as a second element of specificity analogous to the effect of adding a second antibody in a conventional immunoassay.

Cognate SOMAmer-protein complexes were then recaptured on a second set of streptavidin-coated beads using biotin labelled proteins and additionally washed to further remove non-specific SOMAmer-protein interactions. In the next step, denaturing buffer released the SOMAmers from the proteins. The SOMAmers were then hybridized to complementary sequences on a DNA microarray chip and quantified by fluorescence for readout. In the SomaScan assay, fluorescence intensity for each SOMAmer is related to the relative availability of the three-dimensional shape-charge epitope on each protein (the binding site of the SOMAmer reagent) in the original sample. This reflects the concentration of each protein. Median intra- and inter-assay coefficients of variation are ~5% and assay sensitivity is comparable to that of typical immunoassays, with a median lower limit of detection in the femtomolar range.[23] Specificity of the modified aptamer reagents is high.[22]

Full list of SomaScan, version 4 proteins is available in Williams et al.[22]

Conversion of earlier ICD-codes for dementia to ICD-10 codes

ICD-10 codes for dementia were F00, F01, F02, F03, G30, and G31. Earlier ICD-codes for dementia were as follows: 29000, 29010, 29011, 29019, 34791, and 34792 for ICD-8 and 2900A, 2941A, 3310A, 3311A, 3312X, and 4378A for ICD-9.

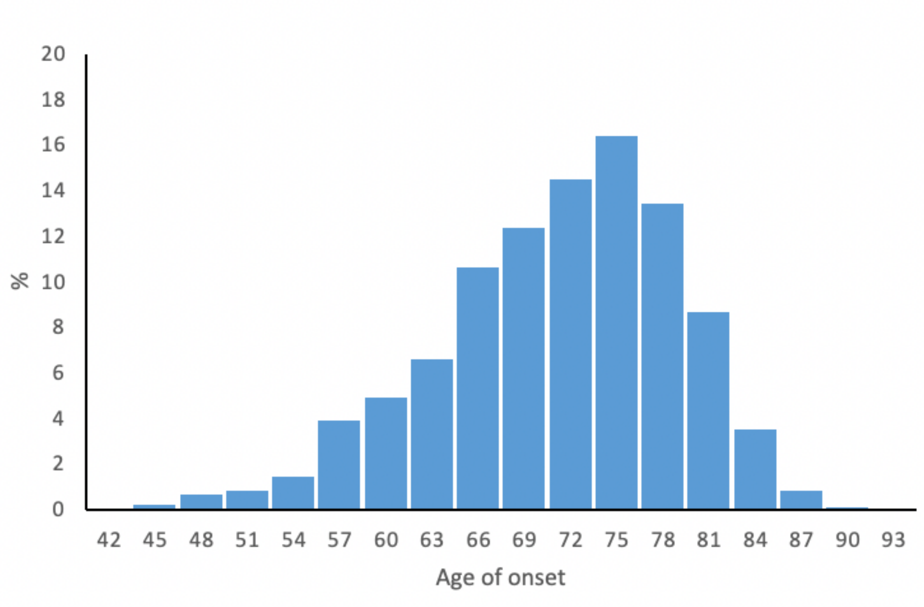

eFigure 2. Distribution of age at dementia diagnosis in main analysis

Study	Cognitive stimulation at work	N(total)	N(dementia)	Hazard ratio (95% confidence interval)
**Gazel**	Low	2281	2	1.00
	Medium	5323	7	1.47 (0.30 - 7.13)
	High	3758	5	1.51 (0.29 - 7.92)
**WOLF N & S**	Low	3007	6	1.00
	medium	4405	10	1.23 (0.45 - 3.40)
	High	2956	9	2.02 (0.72 - 5.72)
**HeSSup**	Low	4168	12	1.00
	medium	7304	27	1.10 (0.56 - 2.17)
	High	4062	11	0.78 (0.35 - 1.78)
**HHS**	Low	1668	13	1.00
	medium	3075	22	0.99 (0.50 - 1.97)
	High	1801	8	0.62 (0.26 - 1.49)
**Whitehall II**	Low	1993	65	1.00
	medium	2778	74	1.00 (0.71 - 1.41)
	High	2704	44	0.73 (0.49 - 1.09)
**FPS**	Low	13326	99	1.00
	medium	23337	161	0.98 (0.76 - 1.26)
	High	10785	53	0.72 (0.52 - 1.01)
**Still Working**	Low	2800	159	1.00
	medium	4502	267	0.96 (0.79 - 1.18)
	High	1863	89	0.78 (0.60 - 1.01)
**Summary Estimate**				
	Low	29243	356	1.00
	medium	50724	568	0.99 (0.86 - 1.13)
	High	27929	219	0.77 (0.65 - 0.92)

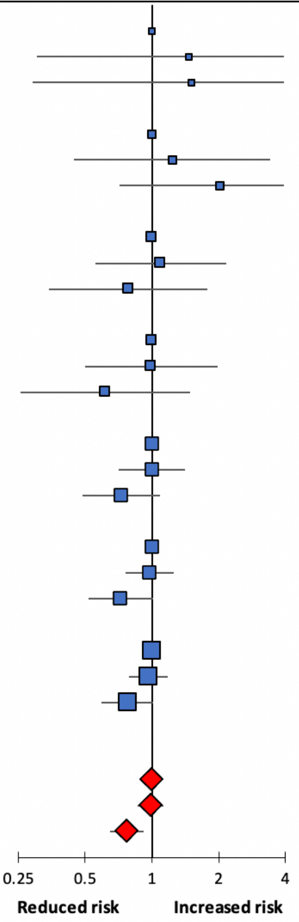

Random effects model
Heterogeneity I^2 = 0%,*

*Hazard ratio adjusted for age and sex.
Heterogeneity in study-specific estimates I2 = 0%, p-heterogeneity = 0.62 for high and 0.99 for medium cognitive stimulation.

eFigure 3. Meta-analysis of the 7 cohort studies on the association between cognitive stimulation at work and risk of dementia.

eTable 12. Association of education with incident dementia in total sample

Adjustment in addition to age and sex	Education	N(total)	N(dementia	HR	95% CI	Attenuation (%)
None	Low	26036	468	1.00		
	Medium	40723	331	0.87	(0.75 - 1.00)	
	High	37171	218	0.66	(0.55 - 0.79)	0 (reference)
Cognitive stimulation	Low	26036	468	1.00		
	Medium	40723	331	0.87	(0.75 - 1.01)	
	High	37171	218	0.68	(0.56 - 0.82)	6.1
Adulthood risk factors*	Low	23845	437	1.00		
	Medium	37678	310	0.90	(0.78 - 1.05)	
	High	34164	195	0.72	(0.59 - 0.87)	20.0
Cardiometabolic diseases†	Low	26025	467	1.00		
	Medium	40717	331	0.88	(0.76 - 1.02)	
	High	37171	218	0.67	(0.56 - 0.81)	5.1
All above	Low	23845	437	1.00		
	Medium	37678	310	0.93	(0.79 - 1.08)	
	High	34164	195	0.75	(0.61 - 0.92)	30.8

* Adjusted for age, sex, cohort and smoking, alcohol consumption, physical inactivity, job strain, obesity, hypertension, and prevalent diabetes at baseline.

† Adjusted for age, sex, cohort and diabetes, coronary heart disease and stroke (prevalent at baseline and incident between baseline and dementia diagnosis).

eTable 13. Association between cognitive stimulation based on job exposure matrix and incident dementia in relation to adjustments and additional covariates (supplementary analysis)(N = 47 445, 313 dementia cases in all models)

Adjustment in addition to age and sex	Hazard ratio per 1SD higher cognitive stimulation (95% confidence interval)		Proportion (%) of effect attenuation
None	0.77	(0.69 - 0.86)	0 (reference)
Education	0.81	(0.69 - 0.96)	20.2
Adulthood risk factors*	0.80	(0.72 - 0.90)	16.5
Prevalent and incident cardiometabolic disease†	0.77	(0.69 - 0.86)	2.0
All above	0.84	(0.71 - 0.99)	33.2
**Additional covariates**			
Social isolation	0.77	(0.69 - 0.86)	1.5
Depression	0.77	(0.69 - 0.86)	-1.0
Traumatic brain injury	0.77	(0.69 - 0.86)	-1.0
Atrial fibrillation	0.77	(0.69 - 0.86)	0.0
**Fully adjusted‡**	0.83	(0.72 - 0.97)	29.1
**Competing risk of death**	0.78	(0.69 - 0.87)	3.0

* Adjusted for age, sex and smoking, alcohol consumption, physical inactivity, job strain, obesity, hypertension, and prevalent diabetes at baseline

† Adjusted for age, sex and diabetes, coronary heart disease and stroke (prevalent at baseline and incident between baseline and dementia diagnosis)

‡ Adjusted for all covariates

A High versus low cognitive stimulation

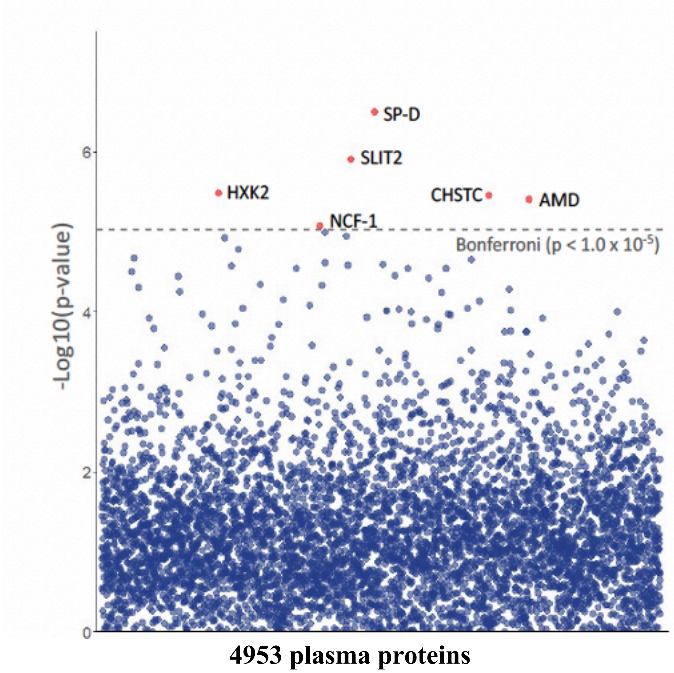

B Medium versus low cognitive stimulation

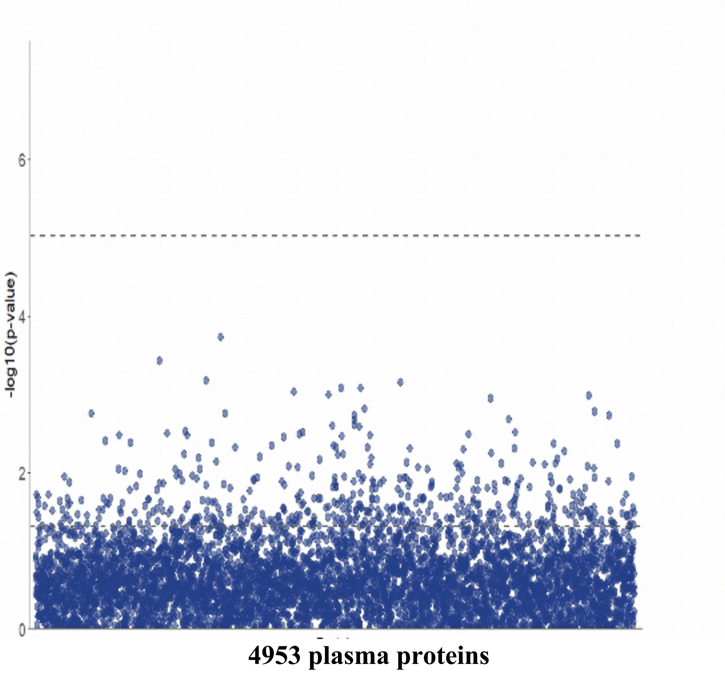

Protein name	UniProt
ulmonary surfactant-associated protein D (SP-D)	P35247
Slit homolog 2 protein (SLIT2)	O94813
Hexokinase-2 (HXK2)	P52789
Carbohydrate sulfotransferase 12 (CHSTC)	Q9NRB3
Peptidyl-glycine alpha-amidating monooxygenase (AMD)	P19021
Neutrophil cytosol factor 1 (NCF-1)	P14598

eFigure 4. A Manhattan plot of plasma proteins for high (A) and medium (B) vs low cognitive stimulation at work. Dashed line shows threshold for Bonferroni-corrected significance.

eTable 14. Multivariable adjusted associations between cognitive stimulation and 3 proteins

Adjustment in addition to age and sex	N (total)	SLIT2 Beta (SE)	SLIT2 P-value	CHSTC Beta (SE)	CHSTC P-value	AMD Beta (SE)	AMD P-value
None	1312	-0.30 (0.06)	0.000001	-0.31 (0.06)	0.0000002	-0.29 (0.06)	0.000001
Education	962	-0.30 (0.08)	0.00007	-0.30 (0.07)	0.00004	-0.28 (0.08)	0.0002
Adulthood risk factors *	1090	-0.32 (0.07)	0.000003	-0.34 (0.07)	0.0000003	-0.29 (0.07)	0.00003
Prevalent and incident cardiometabolic disease†	1293	-0.29 (0.06)	0.000003	-0.32 (0.06)	0.0000001	-0.29 (0.06)	0.000002
Ethnicity	1312	-0.30 (0.06)	0.000001	-0.29 (0.06)	0.000002	-0.27 (0.06)	0.000008
APOE genotype	1114	-0.30 (0.07)	0.000008	-0.26 (0.06)	0.00007	-0.30 (0.07)	0.000003
All above	698	-0.34 (0.10)	0.0003	-0.33 (0.09)	0.0002	-0.32 (0.10)	0.0009

* Adjusted for age, sex and smoking, alcohol consumption, physical inactivity, job strain, obesity, hypertension, and prevalent diabetes at baseline

† Adjusted for age, sex and diabetes, coronary heart disease and stroke (prevalent at baseline and incident between baseline and dementia diagnosis)

eTable 15. Multivariable adjusted associations between 3 proteins and incidence of dementia by cohort

Cohort and protein	Adjustment in addition to age, sex and ethnicity	N (total)	N (dementia)	HR for dementia (95% CI)
**Whitehall II sub-cohort**				
SLIT2	None	2261	106	1.15 (0.94 to 1.40)
	Education	2261	106	1.14 (0.93 to 1.40)
	Adulthood risk factors*	2261	106	1.15 (0.94 to 1.41)
	Cardiometabolic disease†	2261	106	1.14 (0.93 to 1.39)
	All above	2262	107	1.15 (0.94 to 1.41)
	Competing risk of death	2261	106	1.14 (0.92 to 1.40)
AMD	None	2261	106	1.06 (0.87 to 1.28)
	Education	2261	106	1.05 (0.87 to 1.28)
	Adulthood risk factors*	2261	106	1.01 (0.83 to 1.24)
	Cardiometabolic disease†	2261	106	1.05 (0.86 to 1.28)
	All above	2262	107	1.01 (0.83 to 1.24)
	Competing risk of death	2261	106	1.05 (0.85 to 1.28)
CHSTC	None	2261	106	1.10 (0.91 to 1.33)
	Education	2261	106	1.10 (0.91 to 1.33)
	Adulthood risk factors*	2261	106	1.10 (0.91 to 1.33)
	Cardiometabolic disease†	2261	106	1.09 (0.90 to 1.32)
	All above	2262	107	1.07 (0.88 to 1.30)
	Competing risk of death	2261	106	1.08 (0.89 to 1.31)
**ARIC**				
SLIT2	None	11395	1942	1.12 (1.00 to 1.26)
	Education	11395	1942	1.13 (1.01 to 1.27)
	Adulthood risk factors*	11395	1942	1.15 (1.02 to 1.28)
	Cardiometabolic disease†	11395	1942	1.13 (1.01 to 1.27)
	All above	11395	1942	1.16 (1.03 to 1.30)
	Competing risk of death	11395	1942	1.16 (1.04 to 1.30)

AMD	None	11395	1942	1.07 (0.99 to 1.16)
	Education	11395	1942	1.07 (0.99 to 1.16)
	Adulthood risk factors*	11395	1942	1.07 (0.99 to 1.16)
	Cardiometabolic disease†	11395	1942	1.07 (0.98 to 1.16)
	All above	11395	1942	1.05 (0.97 to 1.14)
	Competing risk of death	11395	1942	1.05 (0.97 to 1.14)
CHSTC	None	11395	1942	1.22 (1.05 to 1.41)
	Education	11395	1942	1.20 (1.03 to 1.39)
	Adulthood risk factors*	11395	1942	1.13 (0.97 to 1.31)
	Cardiometabolic disease†	11395	1942	1.19 (1.02 to 1.38)
	All above	11395	1942	1.16 (1.00 to 1.35)
	Competing risk of death	11395	1942	1.16 (1.00 to 1.35)

* Adjusted for age, sex and smoking, alcohol consumption, physical inactivity, job strain, obesity, hypertension, and prevalent diabetes at baseline

† Adjusted for age, sex and diabetes, coronary heart disease and stroke (prevalent at baseline and incident between baseline and dementia diagnosis)

eTable 16. Summary estimates of associations between 3 proteins and incidence of dementia in relation to adjustments

Protein	Adjustment in addition to age, sex and ethnicity	N (total)	N (dementia)	HR for dementia (95% CI)*
SLIT2	None	13 656	2051	1.14 (1.03 to 1.25)
	Education	13 656	2051	1.13 (1.02 to 1.25)
	Adulthood risk factors†	13 656	2051	1.15 (1.04 to 1.27)
	Prevalent and incident cardiometabolic disease‡	13 656	2051	1.13 (1.03 to 1.25)
	All above	14 656	2052	1.16 (1.05 to 1.28)
	Competing risk of death	13 656	2051	1.16 (1.05 to 1.28)
AMD	None	13 656	2051	1.08 (1.00 to 1.16)
	Education	13 656	2051	1.07 (0.99 to 1.15)
	Adulthood risk factors†	13 656	2051	1.06 (0.99 to 1.14)
	Prevalent and incident cardiometabolic disease‡	13 656	2051	1.07 (0.99 to 1.15)
	All above	14 656	2052	1.04 (0.97 to 1.13)
	Competing risk of death	13 656	2051	1.05 (0.97 to 1.13)
CHSTC	None	13 656	2051	1.17 (1.04 to 1.31)
	Education	13 656	2051	1.16 (1.03 to 1.31)
	Adulthood risk factors†	13 656	2051	1.12 (0.99 to 1.26)
	Prevalent and incident cardiometabolic disease‡	13 656	2051	1.15 (1.03 to 1.30)
	All above	14 656	2052	1.13 (1.00 to 1.27)
	Competing risk of death	13 656	2051	1.13 (1.00 to 1.27)

* Hazard ratios are per 1SD higher protein level.

† Adjusted for age, sex and smoking, alcohol consumption, physical inactivity, job strain, obesity, hypertension, and prevalent diabetes at baseline

‡ Adjusted for age, sex and diabetes, coronary heart disease and stroke (prevalent at baseline and incident between baseline and dementia diagnosis)

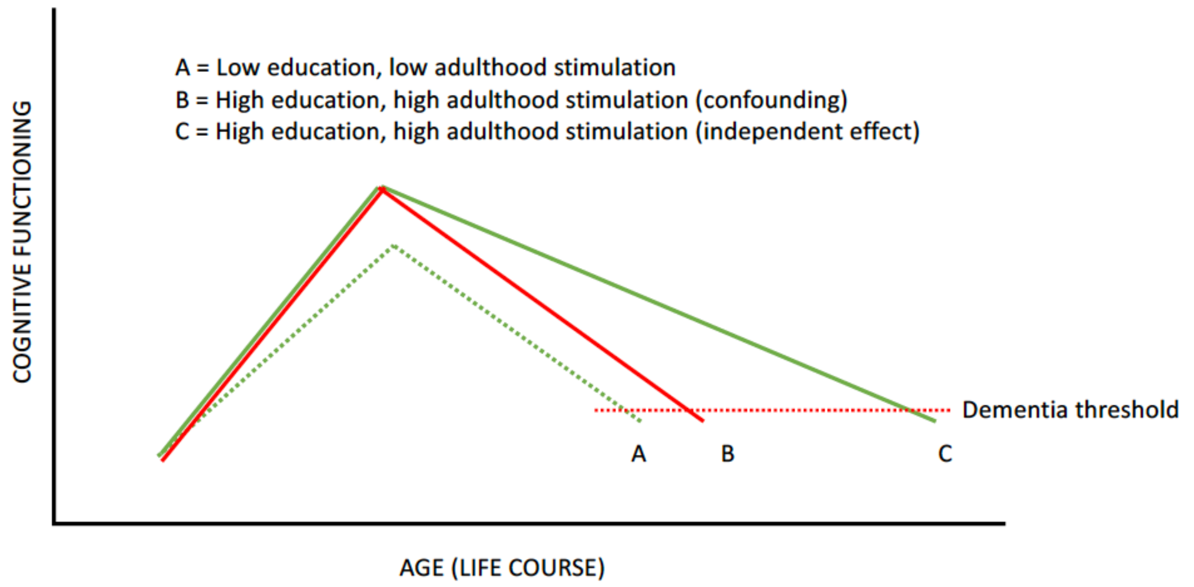

eFigure 5. Cognitive function across the life course according to independent effects of education and adulthood cognitive stimulation and an education-confounded effect of adulthood cognitive stimulation. According to the confounding hypothesis, cognitive stimulation in childhood (i.e. education) tracks into adulthood such that the association of cognitive stimulation in adulthood is completely attributable to the effects of education and thus confounded (trajectory B in the figure). The alternative hypothesis is that the effects of childhood and adulthood cognitive stimulation on dementia risk are additive (trajectory C in the figure). Cognitive function improves in childhood and achieves its peak in early adulthood. Age-related cognitive decline typically starts when people are in their 40s. Early life cognitive stimulation affects the peak level of cognitive function that is achieved (the higher the stimulation, the higher the peak performance). Midlife cognitive stimulation, rather than affecting peak functioning, may reduce later life cognitive decline: the higher the stimulation the more the rate of this decline is reduced.

Statistical code

TABLE 2: Characteristics of participants from the pooled sample

Statistical software: SAS, version 9.4

Code:

```
proc freq data=d3;
     tables sex age60 active3 educ smoke2 alco2 inactive strain2 obese exhypert exdb
db chd stroke;
proc means data=d3;
     var age;
run;
```

FIGURE 2: Crude cumulative hazard of dementia by age and level of cognitive stimulation at work

Statistical software: SAS, version 9.4

Code:

```
proc phreg data=d3;
     model (age age_end)*status_dem(0) = ;
     strata active3;
     baseline out=apu1 survival=survival;
run;
data apu2;
     set apu1;
     rename age_end=time active3=group;
run;
data apu3;
     set apu2;
     by group;
     retain prehazard 0;
     hazard=1-survival;
     if (hazard>. and hazard NE prehazard) or first.group;
     prehazard=hazard;
     keep group time hazard;
data apu4;
     set apu3;
     retain prehazard pregroup 0;
     if group=pregroup then do;
        cumhazard=prehazard; output; end;
     cumhazard=hazard; output;
     pregroup=group;
     prehazard=hazard;
     keep group time cumhazard;
data apu4;
     set apu4;
     cumhazard=100*cumhazard;
run;
proc print data=apu4; ** => Excel figure **;
run;
```

FIGURE 3. Association of cognitive stimulation at work with incident dementia in total sample, subgroups, by study follow-up and in relation to adjustments (step 1)

***MAIN EFFECT, COHORT SPECIFIC ANALYSIS ***

Statistical software: SAS, version 9.4

Code:

```
proc phreg data=d3;
     class active3 (ref='1');
     model futime_dem*status_dem(0)= sex age active3 / rl;
     by study;
     ods output ParameterEstimates=pe CensoredSummary=cs;
     data pe; set pe; if parameter='active3';
     data res; merge pe cs; by study;
     keep study Estimate StdErr ProbChiSq ClassVal0 HazardRatio HRLowerCL HRUpperCL
Total Event;
     proc print data=res;
run;
```

***COMBINING COHORT-SPECIFIC ESTIMATES ***

Statistical software: R, version 4.0

Code:

```
# active3 2 vs 1
library(meta)
labels<-c("FPS","Gazel","HeSSup","HHS","Still Working","WOLF","WH II")
est1<-c  (-0.01929,0.38535,0.09278,-0.00725,-0.03843,0.20986,0.00041)
se1<-c   (0.12776,0.80577,0.34713,0.34991,0.10313,0.51773,0.17554)
met1<-metagen(est1, se1,sm="HR",labels,comb.fixed=FALSE,comb.random=TRUE)
summary(met1)
forest(met1,leftcols="studlab",print.tau2=FALSE)
# active3 3 vs 1
library(meta)
labels<-c("FPS","Gazel","HeSSup","HHS","Still Working","WOLF","WH II")
est1<-c  (-0.32481,0.41378,-0.24496,-0.48580,-0.25268,0.70507,-0.31468)
se1<-c   (0.17032,0.84490,0.41823,0.44953,0.13615,0.53046,0.20532)
met1<-metagen(est1, se1,sm="HR",labels,comb.fixed=FALSE,comb.random=TRUE)
summary(met1)
forest(met1,leftcols="studlab",print.tau2=FALSE)
```

SUBGROUP ANALYSES

Statistical software: SAS, version 9.4

Code:

```
*********************************************************************;
**  pooled data  **;
*********************************************************************;
proc phreg data=d3;
where sex=1;
*where sex=2;
*where age60=0;
*where age60=1;
*where studydik=1;
*where studydik=2;
     class study active3(ref='1');
     model futime_dem*status_dem(0)= sex age study active3 / rl;
*    model futime_dem*status_dem(0)= sex age study active3 sex*active3 / rl;
 run;
** Length of follow-up: <10/10+ **;
proc freq data=d3;
     tables active3*(status_demA status_demB) / nopercent nocol norow;
```

```
run;
proc phreg data=d3;
     class study active3(ref='1');
     model futime_demA*status_demA(0)= sex age study active3 / rl;
*    model futime_demB*status_demB(0)= sex age study active3 / rl;
run;
* proportionality test *;
data d4; set d3;
act2=0; if active3=2 then act2=1;
act3=0; if active3=3 then act3=1;
run;
proc phreg data=d4;
     class study;
     model futime_dem*status_dem(0)= sex age study act2 act3 timeact2 timeact3 / rl;
     if futime_dem<10 then timeact2=0;
     if futime_dem<10 then timeact3=0;
     if futime_dem>=10 then timeact2=act2;
     if futime_dem>=10 then timeact3=act3;
     proportionality_test: test timeact2, timeact3;
run;
** Early onset (<65) / Late onset (65+) **;
data t3; set d3; ** <65 **;
     if age<65;
     if futime_dem>(65-age) then do; futime_dem=65-age; status_dem=0; end;
proc phreg data=t3;
     class study active3(ref='1');
     model futime_dem*status_dem(0)= sex age study active3 / rl;
run;
data tt3; set d3; ** 65+ **;
     if futime_dem<=(65-age) then delete;
     futime_dem=min(futime_dem,futime_dem-(65-age));
     if age<65 then age=65;
proc phreg data=tt3;
     class study active3(ref='1');
     model futime_dem*status_dem(0)= sex age study active3 / rl;
run;
* proportionality test *;
proc phreg data=d4;
     class study;
     model (age age_end)*status_dem(0)= sex study act2 act3 timeact2 timeact3 / rl;
     if age_end<65 then timeact2=0;
     if age_end<65 then timeact3=0;
     if age_end>=65 then timeact2=act2;
     if age_end>=65 then timeact3=act3;
     proportionality_test: test timeact2, timeact3;
run;
** Alzheimer / other dementia **;
proc phreg data=d3; where study IN ('FPS','HEA','Gaz','STW','WO');
     class study active3(ref='1');
     model futime_dem*status_alz(0)= sex age study active3 / rl;
*    model futime_dem*status_other(0)= sex age study active3 / rl;
run;
data test;
     b1=-0.33169;
     se1=0.11571;
     b2=0.08147;
     se2=0.18716;
     chi2=((b1-b2)**2)/(se1**2+se2**2);
     df=1;
     p=1-probchi(chi2,df);
proc print data=test;
     var chi2 df p;
run;
** Adjusted for education **;
proc phreg data=d3;
     class study educ(ref='1') active3(ref='1');
     model futime_dem*status_dem(0)= sex age study educ active3 / rl;
run;
** Adjusted for time-dependent CMD **;
```

```
proc phreg data=d4;
     class study active3(ref='1');
     model futime_dem*status_dem(0)= sex age study timedep_db timedep_chd
timedep_stroke active3 / rl;
     if futime_dem<=exptime_db then timedep_db=0; else timedep_db=1;
     if futime_dem<=exptime_chd then timedep_chd=0; else timedep_chd=1;
     if futime_dem<=exptime_stroke then timedep_stroke=0; else timedep_stroke=1;
run;
** Competing risk of death **;
proc phreg data=d3;
     class study active3(ref='1');
     model futime_dem*status_dem3d(0)= sex age study active3 / eventcode=1 rl;
run;
** Incidence per 10,000 person-years **;
proc means data=d3 nway noprint;
where sex=1;
*where sex=2;
*where age60=0;
*where age60=1;
*where studydik=1;
*where studydik=2;
     var status_dem futime_dem;
     class active3;
     output out=s1 sum=;
data ss1;
     set s1;
     incid=(status_dem/futime_dem)*10000;
proc print data=ss1;
     id active3;
     var _FREQ_ status_dem incid;
run;
```

IMPUTED ANALYSES FOR ADJUSTMENTS

Statistical software: STATA, version 16·1 MP; Stata Corp, College Station, TX, USA

Code:

```
*Figure 3, imputed data in step 1

stset futime_dem, failure(status_dem==1) id(id2)
misschk age sex exdb educ exhypert db chd stroke inactive obese smoke2 alco2 active3
strain2 educ_act educ_act_sum study_no study_year

set seed 123
smcfcs stcox age sex  educ exdb exhypert db chd stroke inactive obese smoke2 alco2
active3 strain2 study_no study_year, logit(exhypert exdb db inactive obese smoke2
alco2) ologit(educ)   m(30) iter(5) rseed(123)

mi stset futime_dem, failure(status_dem ==1) id(id2)
putexcel set "Results.xlsx", sheet("Figure3") modify

*Education
local i = 1
mi estimate:  stcox age sex i.active3 i.educ i.study_no
mi estimate, hr
mat m =r(table)
putexcel F`i' = m[1,4]
putexcel G`i' = m[5,4]
putexcel H`i' = m[6,4]
putexcel M`i' = m[4,4]
local ++i
putexcel F`i' = m[1,5]
putexcel G`i' = m[5,5]
putexcel H`i' = m[6,5]
putexcel M`i' = m[4,5]

*Adulthood risk factors
```

```
local i = 4
mi estimate:  stcox age sex i.active3 exhypert exdb inactive obese smoke2 alco2
strain2 i.study_no
mi estimate, hr
mat m =r(table)
putexcel F`i' = m[1,4]
putexcel G`i' = m[5,4]
putexcel H`i' = m[6,4]
putexcel M`i' = m[4,4]
local ++i
putexcel F`i' = m[1,5]
putexcel G`i' = m[5,5]
putexcel H`i' = m[6,5]
putexcel M`i' = m[4,5]

*Cardiometabolic risk factors
local i = 6
mi estimate:  stcox age sex i.active3 db chd stroke i.study_no
mi estimate, hr
mat m =r(table)
putexcel F`i' = m[1,4]
putexcel G`i' = m[5,4]
putexcel H`i' = m[6,4]
putexcel M`i' = m[4,4]
local ++i
putexcel F`i' = m[1,5]
putexcel G`i' = m[5,5]
putexcel H`i' = m[6,5]
putexcel M`i' = m[4,5]
```

TABLE 3. Proteins associated with cognitive stimulation after controlling for multiple testing (step 2)*

LOGISTIC REGRESSION FOR THE ASSOCIATIONS OF PROTEINS WITH COGNITIVE STIMULATION CATEGORY (HIGH VS LOW; MEDIUM VS LOW)

Statistical software: STATA, version 16·1 MP; Stata Corp, College Station, TX, USA

Code:

```
*Table 3, step 2
stset exitdate_dem_19, failure(dementia_ad19==1) enter(time start) origin(start)
scale(365.25) id(ID_OUT)
putexcel set "Results.xlsx", sheet("Table3") modify

local i = 1
foreach protein in invr_norm_SLIT2 invr_norm_CHSTC invr_norm_AMD invr_norm_SPD
invr_norm_HXK2 invr_norm_NCF1 {
logistic js1 age_s5 sex `protein' i.ETHN_DS, coef
mat m =r(table)
putexcel F`i' = m[1,3]
putexcel G`i' = m[2,3]
putexcel H`i' = m[4,3]
local ++i
}

local i = 1
foreach protein in invr_norm_SLIT2 invr_norm_CHSTC invr_norm_AMD invr_norm_SPD
invr_norm_HXK2 invr_norm_NCF1 {
logistic js_2 age_s5 sex `protein' i.ETHN_DS, coef
mat m =r(table)
putexcel J`i' = m[1,3]
putexcel K`i' = m[2,3]
putexcel L`i' = m[4,3]
local ++i
}
```

TABLE 4. Association between cognitive stimulation-related plasma proteins and incident dementia in relation to adjustments (step 3)

Statistical software: STATA, version 16·1 MP; Stata Corp, College Station, TX, USA

Code:
```
* Table4, step 3
* Check convergence of imputation model

misschk age_s5 sex exdb exhypert obese smoke2 alco2 inactive strain2 apoe_bin ETHN_DS
educ db chd stroke
stset exitdate_dem_19, failure(dementia_ad19==1) enter(time start) scale(365.25)
id(ID_OUT)
smcfcs stcox age_s5 sex exdb exhypert obese smoke2 alco2 inactive strain2 apoe_bin
i.ETHN_DS i.educ db chd stroke  invr_norm_SLIT2 invr_norm_CHSTC invr_norm_AMD,
logit(exdb exhypert obese smoke2 alco2 inactive strain2 apoe_bin db chd stroke)
ologit(educ)   m(1) iter(100) rseed(123) chainonly savetrace(Trace)

use Trace, clear
tsset iter
graph drop _all
tsline est2educ, name(gr1) nodraw
tsline est3educ, name(gr2) nodraw
tsline estapoe_bin, name(gr3) nodraw
tsline eststrain2, name(gr4) nodraw
tsline estinvr_norm_SLIT2, name(gr5) nodraw
tsline estinvr_norm_CHSTC, name(gr6) nodraw
tsline estinvr_norm_AMD, name(gr7) nodraw
tsline estage_s5, name(gr8) nodraw
graph combine  gr1 gr2 gr3 gr4 gr5 gr6 gr7 gr8, title(Trace plots) rows(4)

* Run imputation model
stset exitdate_dem_19, failure(dementia_ad19==1) enter(time start) scale(365.25)
id(ID_OUT)
smcfcs stcox age_s5 sex exdb exhypert obese smoke2 alco2 inactive strain2 apoe_bin
i.ETHN_DS i.educ db chd stroke  invr_norm_SLIT2 invr_norm_CHSTC invr_norm_AMD,
logit(exdb exhypert obese smoke2 alco2 inactive strain2 apoe_bin db chd stroke)
ologit(educ)  m(30) iter(5) rseed(123)
*Categorical/binary variables
midiagplots  educ, m(1/10) combine plottype(histogram)
midiagplots  educ, m(11/20) combine plottype(histogram)
midiagplots  educ, m(21/30) combine plottype(histogram)
midiagplots  strain2, m(1/10) combine plottype(histogram)
midiagplots  strain2, m(11/20) combine plottype(histogram)
midiagplots  strain2, m(21/30) combine plottype(histogram)
midiagplots  inactive, m(1/10) combine plottype(histogram)
midiagplots  inactive, m(11/20) combine plottype(histogram)
midiagplots  inactive, m(21/30) combine plottype(histogram)
mi stset exitdate_dem_19, failure(dementia_ad19==1) enter(time start) scale(365.25)
id(ID_OUT)
putexcel set "Results.xlsx", sheet("Table4") modify

local i = 3
foreach protein in invr_norm_SLIT2 invr_norm_CHSTC invr_norm_AMD {
mi estimate: stcox age_s5 sex `protein' i.educ i.ETHN_DS
mi estimate, hr
mat m =r(table)
putexcel E`i' = m[1,3]
putexcel F`i' = m[5,3]
putexcel G`i' = m[6,3]
putexcel H`i' = m[4,3]
local ++i
}

local i = 8
foreach protein in invr_norm_SLIT2 invr_norm_CHSTC invr_norm_AMD {
mi estimate: stcox age_s5 sex `protein' exdb exhypert obese smoke2 alco2 inactive
strain2 i.ETHN_DS
```

```
mi estimate, hr
mat m =r(table)
putexcel E`i' = m[1,3]
putexcel F`i' = m[5,3]
putexcel G`i' = m[6,3]
putexcel H`i' = m[4,3]
local ++i
}

local i = 13
foreach protein in invr_norm_SLIT2 invr_norm_CHSTC invr_norm_AMD {

mi estimate: stcox age_s5 sex `protein' db chd stroke i.ETHN_DS
mi estimate, hr
mat m =r(table)
putexcel E`i' = m[1,3]
putexcel F`i' = m[5,3]
putexcel G`i' = m[6,3]
putexcel H`i' = m[4,3]
local ++i
}

stset exitdate_dem_19, failure(cens_comp==1) enter(time start) scale(365.25)
id(ID_OUT)

smcfcs compet age_s5 sex exdb exhypert obese smoke2 alco2 inactive strain2 apoe_bin
i.ETHN_DS i.educ db chd stroke   invr_norm_SLIT2 invr_norm_CHSTC invr_norm_AMD,
logit(exdb exhypert obese smoke2 alco2 inactive strain2 apoe_bin db chd stroke)
ologit(educ)  m(30) iter(5) rseed(123) failure(cens_comp) time(exitdate_dem_19)
enter(start)
mi stset exitdate_dem_19, failure(cens_comp==1) enter(time start) scale(365.25)
id(ID_OUT)
putexcel set "Results.xlsx", sheet("Table4") modify
local i = 18
foreach protein in  invr_norm_SLIT2 invr_norm_CHSTC invr_norm_AMD {
mi estimate:  stcrreg age_s5 sex `protein' i.ETHN_DS, compete(cens_comp==2)
mi estimate, hr
mat m =r(table)
putexcel E`i' = m[1,3]
putexcel F`i' = m[5,3]
putexcel G`i' = m[6,3]
putexcel H`i' = m[4,3]
local ++i
}
```

FIGURE 4. Association of life course cognitive stimulation with incident dementia (a post hoc analysis)

Statistical software: STATA, version 16·1 MP; Stata Corp, College Station, TX, USA

Code:

```
stset futime_dem, failure(status_dem==1) id(id2)
smcfcs stcox age sex exdb exhypert db chd stroke inactive obese smoke2 alco2 active3
strain2 educ_act  study_no study_year, logit(exdb exhypert db inactive obese smoke2
alco2) ologit(educ_act)  m(30) iter(5) rseed(123)
putexcel set "Results.xlsx", sheet("Figure4") modify
local i = 1
mi estimate:  stcox age sex i.educ_act i.study_no
mi estimate, hr
mat m =r(table)
putexcel F`i' = m[1,4]
putexcel G`i' = m[5,4]
putexcel H`i' = m[6,4]
putexcel M`i' = m[4,4]
local ++i
putexcel F`i' = m[1,5]
putexcel G`i' = m[5,5]
putexcel H`i' = m[6,5]
putexcel M`i' = m[4,5]
```

```
local ++i
putexcel F`i' = m[1,6]
putexcel G`i' = m[5,6]
putexcel H`i' = m[6,6]
putexcel M`i' = m[4,6]
```

References

1. Kivimaki M, Kawachi I. Need for more individual-level meta-analyses in social epidemiology: example of job strain and coronary heart disease. *American journal of epidemiology* 2013;177(1):1-2. doi: kws407 [pii]
10.1093/aje/kws407 [published Online First: 2012/11/13]
2. Kivimaki M, Singh-Manoux A, Ferrie JE, et al. Post hoc decision-making in observational epidemiology--is there need for better research standards? *International journal of epidemiology* 2013;42(2):367-70. [published Online First: 2013/04/10]
3. Fransson EI, Nyberg ST, Heikkila K, et al. Comparison of alternative versions of the job demand-control scales in 17 European cohort studies: the IPD-Work consortium. *BMC Public Health* 2012;12:62. doi: 1471-2458-12-62 [pii] 10.1186/1471-2458-12-62 [published Online First: 2012/01/24]
4. Kivimaki M, Nyberg ST, Batty GD, et al. Job strain as a risk factor for coronary heart disease: a collaborative meta-analysis of individual participant data. *Lancet* 2012;380(9852):1491-7. [published Online First: 2012/09/18]
5. Heikkila K, Nyberg ST, Theorell T, et al. Work stress and risk of cancer: meta-analysis of 5700 incident cancer events in 116,000 European men and women. *Bmj* 2013;346:f165. [published Online First: 2013/02/09]
6. Fransson EI, Nyberg ST, Heikkila K, et al. Job strain and the risk of stroke: an individual-participant data meta-analysis. *Stroke* 2015;46(2):557-9. doi: 10.1161/STROKEAHA.114.008019
7. Nyberg ST, Fransson EI, Heikkilä K, et al. Job strain as a risk factor for type 2 diabetes: A pooled analysis of 124 808 men and women. *Diabetes care* 2014;37(8):2268-75.
8. Heikkila K, Madsen IE, Nyberg ST, et al. Job strain and the risk of severe asthma exacerbations: a meta-analysis of individual-participant data from 100 000 European men and women. *Allergy* 2014;69(6):775-83. doi: 10.1111/all.12381 [published Online First: 2014/04/15]
9. Heikkila K, Madsen IE, Nyberg ST, et al. Job strain and COPD exacerbations: an individual-participant meta-analysis. *The European respiratory journal* 2014;44(1):247-51. [published Online First: 2014/04/04]
10. Kivimaki M, Pentti J, Ferrie JE, et al. Work stress and risk of death in men and women with and without cardiometabolic disease: a multicohort study. *The lancet Diabetes & endocrinology* 2018;6(9):705-13. doi: 10.1016/S2213-8587(18)30140-2 [published Online First: 2018/06/10]
11. The_ investigators. The Atherosclerosis Risk in Communities (ARIC) Study: Design and objectives. *American journal of epidemiology* 1989;129:687-702.
12. Kivimaki M, Lawlor DA, Davey Smith G, et al. Socioeconomic position, co-occurrence of behavior-related risk factors, and coronary heart disease: the Finnish Public Sector study. *American journal of public health* 2007;97(5):874-9. doi: AJPH.2005.078691 [pii]10.2105/AJPH.2005.078691 [published Online First: 2007/03/31]
13. Goldberg M, Leclerc A, Bonenfant S, et al. Cohort profile: the GAZEL Cohort Study. *International journal of epidemiology* 2007;36(1):32-9.

14. Korkeila K, Suominen S, Ahvenainen J, et al. Non-response and related factors in a nation-wide health survey. *European journal of epidemiology* 2001;17(11):991-9. [published Online First: 2002/10/17]
15. Lahelma E, Aittomaki A, Laaksonen M, et al. Cohort profile: the Helsinki Health Study. *International journal of epidemiology* 2013;42(3):722-30. doi: 10.1093/ije/dys039 [published Online First: 2012/04/03]
16. Kalimo S, Toppinen S. Organizational well-being: ten years of research and development: in a forest industry corporation. In: Kompier M, Cooper C, eds. Preventing Stress, Improving Productivity: European Case Studies in the Workplace. London: Routledge 1999:52-85.
17. Marmot M, Brunner E. Cohort Profile: the Whitehall II study. *International journal of epidemiology* 2005;34(2):251-6.
18. Alfredsson L, Hammar N, Fransson E, et al. Job strain and major risk factors for coronary heart disease among employed males and females in a Swedish study on work, lipids and fibrinogen. *Scandinavian journal of work, environment & health* 2002;28(4):238-48. doi: 671 [pii] [published Online First: 2002/08/30]
19. Peter R, Alfredsson L, Hammar N, et al. High effort, low reward, and cardiovascular risk factors in employed Swedish men and women: baseline results from the WOLF Study. *Journal of epidemiology and community health* 1998;52(9):540-7.
20. Karasek RA. Job demands, job decision latitude and mental strain: implications for job redesign. *Administrative Science Quartely* 1979;24:285-307.
21. Karasek RA, Theorell T. Stress, productivity and reconstruction of working life. New York: Basic Books 1990.
22. Williams SA, Kivimaki M, Langenberg C, et al. Plasma protein patterns as comprehensive indicators of health. *Nat Med* 2019;25(12):1851-57. doi: 10.1038/s41591-019-0665-2 [published Online First: 2019/12/04]
23. Kim CH, Tworoger SS, Stampfer MJ, et al. Stability and reproducibility of proteomic profiles measured with an aptamer-based platform. *Sci Rep* 2018;8(1):8382. doi: 10.1038/s41598-018-26640-w [published Online First: 2018/06/01]
24. Candia J, Cheung F, Kotliarov Y, et al. Assessment of Variability in the SOMAscan Assay. *Sci Rep* 2017;7(1):14248. doi: 10.1038/s41598-017-14755-5 [published Online First: 2017/10/29]
25. Gold L, Ayers D, Bertino J, et al. Aptamer-based multiplexed proteomic technology for biomarker discovery. *PloS one* 2010;5(12):e15004. doi: 10.1371/journal.pone.0015004 [published Online First: 2010/12/18]
