# Supplementary material for: Cognitive stimulation in the workplace, plasma proteins, and risk of dementia: three analyses of population cohort studies
Source: BMJ. 2021 Aug 19;374:n1804. doi: 10.1136/bmj.n1804 (PMC8372196; doi:10.1136/bmj.n1804)
Supplement: Supplementary file 2 — Supplementary information: figure showing details of the three analyses [file kivm064249.wf1.pdf]

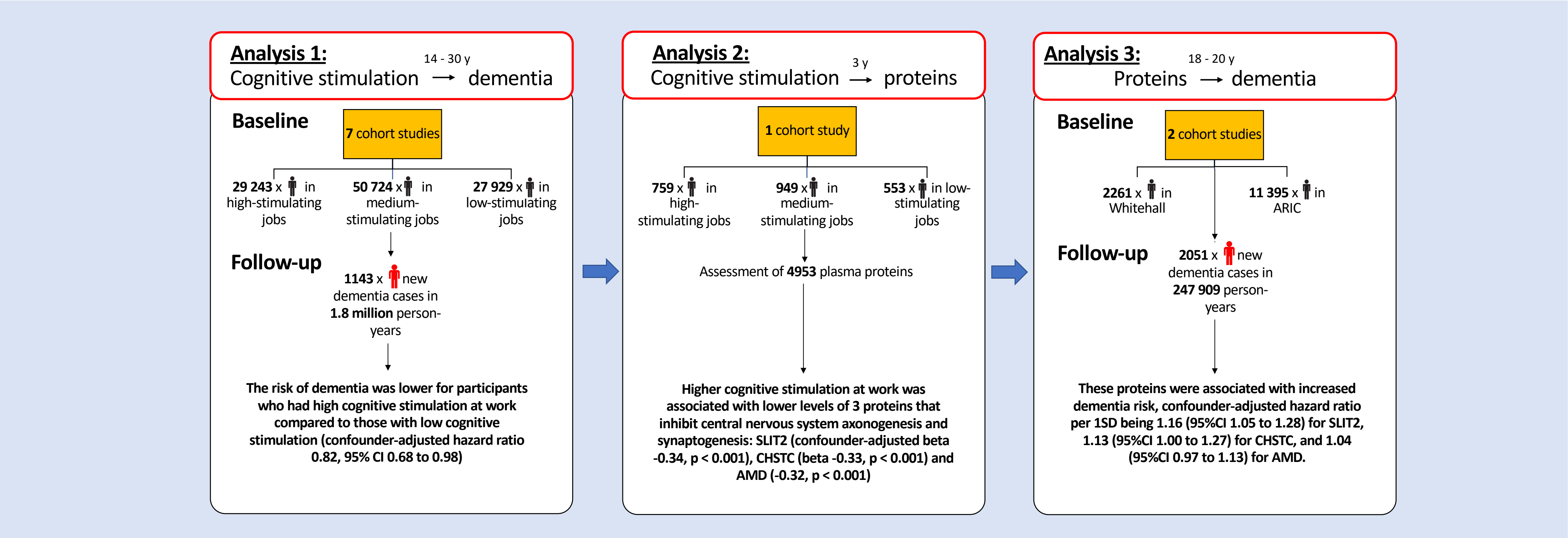
Analysis 1:
Cognitive stimulation → dementia
14 - 30 y
Baseline
7 cohort studies
29 243 x in high-stimulating jobs
50 724 x in medium-stimulating jobs
27 929 x in low-stimulating jobs
Follow-up
1143 x new dementia cases in 1.8 million person-years
The risk of dementia was lower for participants who had high cognitive stimulation at work compared to those with low cognitive stimulation (confounder-adjusted hazard ratio 0.82, 95% CI 0.68 to 0.98)
Analysis 2:
Cognitive stimulation → proteins
3 y
1 cohort study
759 x in high-stimulating jobs
949 x in medium-stimulating jobs
553 x in low-stimulating jobs
Assessment of 4953 plasma proteins
Higher cognitive stimulation at work was associated with lower levels of 3 proteins that inhibit central nervous system axonogenesis and synaptogenesis: SLIT2 (confounder-adjusted beta -0.34, p < 0.001), CHSTC (beta -0.33, p < 0.001) and AMD (-0.32, p < 0.001)
Analysis 3:
Proteins → dementia
18 - 20 y
Baseline
2 cohort studies
2261 x in Whitehall
11 395 x in ARIC
Follow-up
2051 x new dementia cases in 247 909 person-years
These proteins were associated with increased dementia risk, confounder-adjusted hazard ratio per 1SD being 1.16 (95%CI 1.05 to 1.28) for SLIT2, 1.13 (95%CI 1.00 to 1.27) for CHSTC, and 1.04 (95%CI 0.97 to 1.13) for AMD.
